# Surveillance system assessment in Guinea: Training needed to strengthen data quality and analysis, 2016

**DOI:** 10.1371/journal.pone.0234796

**Published:** 2020-06-25

**Authors:** Doreen Collins, Sarah Rhea, Boubacar Ibrahima Diallo, Mariama Boubacar Bah, Facinet Yattara, Rachelle Goman Keleba, Pia D. M. MacDonald

**Affiliations:** 1 RTI International, Research Triangle Park, North Carolina, United States of America; 2 RTI International, Conakry, Guinea; 3 Guinea Ministry of Health, Conakry, Guinea; 4 International Organization for Migration, Boffa, Guinea; 5 Department of Epidemiology, Gillings School of Global Public Health, University of North Carolina, Chapel Hill, North Carolina, United States of America; Tulane University, UNITED STATES

## Abstract

The 2014–2016 Ebola virus disease outbreak revealed the fragility of the Guinean public health infrastructure. As a result, the Guinean Ministry of Health is collaborating with international partners to improve compliance with the International Health Regulations and work toward the Global Health Security Agenda goals, including enhanced case- and community-based disease surveillance. We assessed the case-based disease surveillance system during October 1, 2015–March 31, 2016, in the Boffa prefecture of Guinea. We conducted onsite interviews with public health staff at the peripheral (health center), middle (prefectural), and central (Ministry of Health) levels of the public health system to document leadership structure; methods for maintaining case registers and submitting weekly case reports; disease surveillance feedback; data analysis; and baseline surveillance information on four epidemic-prone diseases (cholera, meningococcal meningitis, measles, and yellow fever). The surveillance system was simple and paper-based at health centers and computer spreadsheet–based at the prefectural and central levels. Surveillance feedback to stakeholders at all levels was infrequent. Data analysis activities were minimal at the peripheral levels and progressively more robust at the prefectural and central levels. Reviewing the surveillance reports from Boffa during the study period, we observed zero reported cases of the four epidemic-prone diseases in the weekly reporting from the peripheral to the central level. Similarly, the national District Health Information System 2 had no reported cases of the four diseases in Boffa but did indicate reported cases among all four neighboring prefectures. Based on the assessment findings, which suggest low sensitivity of the case-based disease surveillance system in Boffa, we recommend additional training and support to improve surveillance data quality and enhance Guinean public health workforce capacity to use these data.

## 1. Introduction

The 2014–2016 Ebola virus disease (EVD) outbreak in West Africa was the largest in history, with over 28,600 reported cases [1]. During the outbreak, widespread EVD transmission occurred in Guinea, Liberia, and Sierra Leone. Among these three countries, Guinea reported the fewest EVD cases and related deaths but experienced the highest case fatality rate [1]. Although the outbreak originated in rural Guinea through a single introduction of the virus into the human population, EVD transmission rapidly spread across national borders, highlighting a lack of capacity to prevent, detect, and respond to emerging infectious disease threats in time to prevent regional and global epidemics [2]. The health systems, including the public health infrastructures, of these countries were weak and unprepared to mitigate widespread disease transmission [3]. To address this challenge and prepare for future disease outbreaks, the Guinean Ministry of Health continues to enhance the public health system to comply with the International Health Regulations (IHR) and work toward the Global Health Security Agenda (GHSA) goals, including strengthened disease surveillance and community-level public health emergency response [4, 5].

To comprehensively meet IHR requirements and ensure a rapid response to acute public health events like EVD, a nation’s indicator- and event-based surveillance should yield high-quality data that are quickly reported to authorities who can take effective action [4]. Case-based disease surveillance, a type of indicator-based surveillance, is the primary method of disease reporting in countries with robust public health infrastructures [6]. In hard-to-reach areas like rural Guinea, where access to basic health care is limited, implementation of community-based surveillance, a type of event-based surveillance, can allow early notification and timely response to disease outbreaks [7]. Community-based surveillance involves reports, stories, rumors, and other unstructured information about health events that could be a public health risk [6].

The Guinean Ministry of Health has identified specific communicable and noncommunicable diseases and conditions or events that are the greatest burden on the health of the nation and are consequently priorities for disease surveillance [8–10]. According to the principles of Integrated Disease Surveillance and Response (IDSR), epidemic-prone diseases in Guinea are a subset of these priority diseases that have high potential to cause serious global health impact because of their ability to spread rapidly internationally [10]. The burden of these epidemic-prone diseases, specifically cholera, meningococcal meningitis, measles, yellow fever, dengue, and viral hemorrhagic fever, in Guinea was not well-documented at the prefecture level prior to District Health Information System 2 (DHIS2) implementation in 2017. According to the most recent data available through the World Health Organization’s (WHO’s) Global Health Observatory, Guinea had 3 reported cases of cholera in 2011, 480 reported cases of meningitis in 2013 (most commonly identified during the dry season from December through June), 2 reported cases of yellow fever in 2014, and 243 reported cases of meningitis in 2015 [8, 10, 11].

In 2015 during the EVD outbreak, the Guinean Ministry of Health began reinforcing support for community-based surveillance across the country with assistance from international partners. A goal of these efforts was to expand community-level capacity for epidemic-prone disease case identification and elevation to authorities who could further investigate. Beginning in March 2016, we were among the partners providing technical support to enhance community-based surveillance activities in Boffa, a rural Guinean coastal prefecture selected by the Guinean Ministry of Health for this work. Boffa has a history of cholera epidemics [12]. With an estimated population of over 200,000, Boffa is one of the five prefectures of the administrative region of Boké [13].

While initiating our technical support efforts in March 2016, we sought relevant baseline information on the existing case-based disease surveillance system in Guinea and on recent prefectural-specific case counts of epidemic-prone diseases. We planned to use this baseline information to target community-based surveillance strengthening activities and, eventually, measure the impact of our efforts. However, information on the existing case-based disease surveillance system in Guinea was not readily available.

Therefore, to inform our technical assistance activities, we systematically documented and assessed the case-based disease surveillance system in Boffa for the time period October 1, 2015–March 31, 2016, in collaboration with the Guinean Ministry of Health. We describe our assessment findings, focused on the surveillance system’s operations, resources, and attributes (i.e., simplicity, data quality). We discuss how the assessment can be used to inform community-based surveillance support efforts in Boffa to further the Guinean Ministry of Health’s mission of strengthening disease surveillance throughout the country.

## 2. Methods

This work was determined to be exempt from human subjects’ review by the RTI International Institutional Review Board. Data were analyzed anonymously. We used the U.S. Centers for Disease Control and Prevention (CDC) guidelines for surveillance system evaluations to assess the case-based disease surveillance system in Boffa for the time period October 1, 2015–March 31, 2016. To initiate this evaluation, we obtained support from stakeholders and reviewed documents that describe the Guinean public health system (e.g., types of public and private clinical health care facilities and their roles in disease surveillance) [14, 15]. With partners, we identified critical questions about the surveillance system and learned the types of information that would be most useful to stakeholders. This guided our assessment to focus on the surveillance system’s operations, resources, and attributes (i.e., simplicity, data quality). Subsequently, we developed questionnaires to conduct in-depth interviews with key informants at sites throughout the health system [16].

The key informant questionnaires covered the following topics: leadership structure at the site and role of the interviewee; methods for maintaining a case register and submitting weekly case reports; outbreak documentation; disease surveillance feedback from other health system levels; data analysis capabilities; status of community-based surveillance implementation; and baseline case-based disease surveillance information on four epidemic-prone diseases (cholera, meningococcal meningitis, measles, and yellow fever) during the time period October 1, 2015–March 31, 2016 (S1 and S2 Files). These four epidemic-prone diseases were chosen considering the epidemic potential of each disease and the number of expected cases during the period based on available national data [8, 10, 11]. Viral hemorrhagic fever was excluded from the assessment because alternative data collection and reporting methods were used for this condition during the EVD outbreak of 2014–2016.

System simplicity was assessed by reviewing the disease-reporting structure, resources used, and ease of operation. Data quality was assessed by evaluating the completeness (i.e., reports were complete) of case recording and reporting during October 1, 2015–March 31, 2016. We documented the structure of the Guinean health system, as related to the case-based disease surveillance system in the Boffa prefecture and from the perspective of stakeholders, at four health care and public health system levels: (1) rural health centers, (2) prefecture hospital, (3) prefectural health department, and (4) Guinean Ministry of Health.

**Fig 1:**
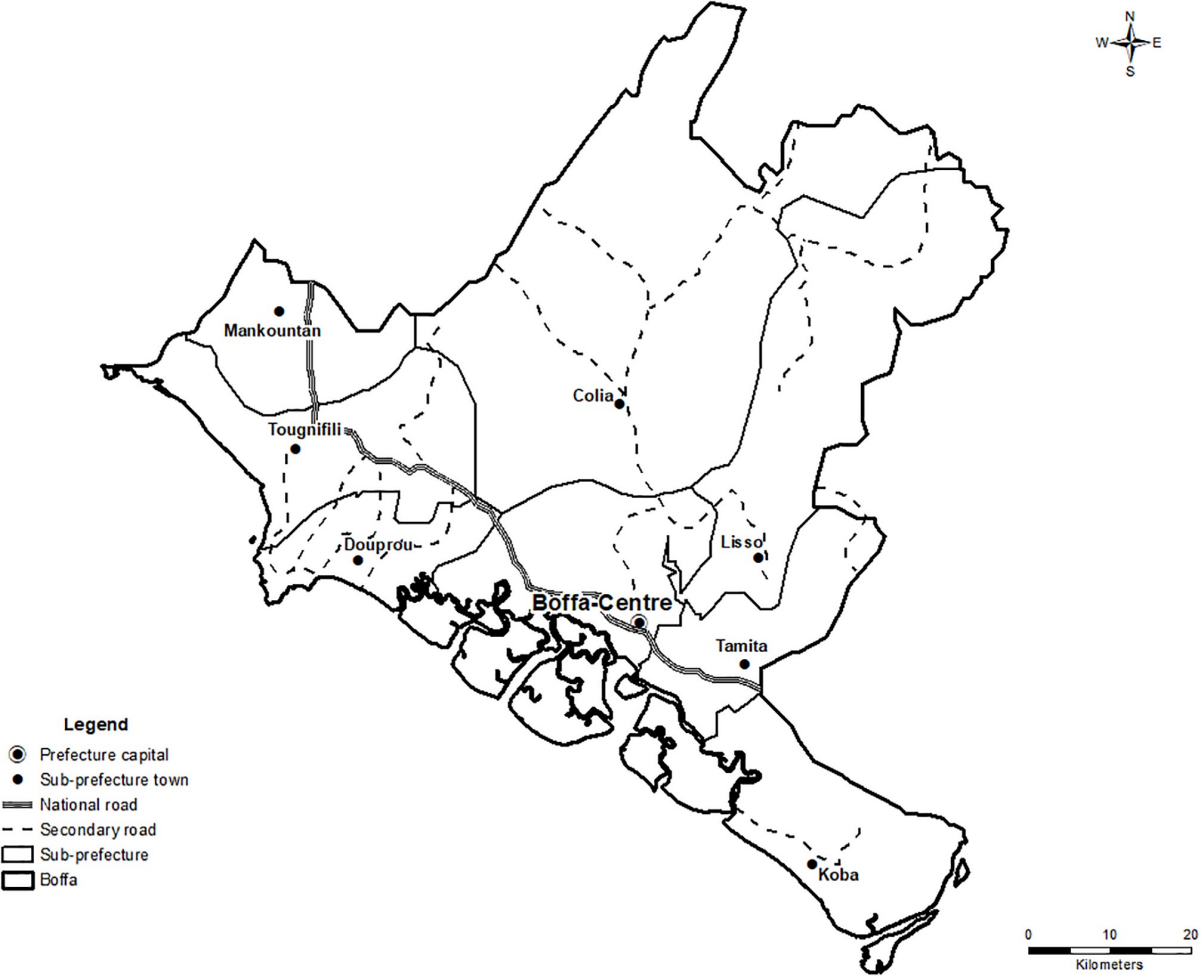
Administrative map of Boffa (carte administrative de Boffa), Guinea, including eight rural health centers.

We assembled a field team of three local, francophone interviewers with experience in surveillance gained during the EVD outbreak response and conducted a single training that included a review of the purpose of a surveillance system assessment, interviewing techniques, data transcription methods, and an orientation to the questionnaires. We did not conduct a formal pre-test assessment of the questionnaires. During September 26–29, 2016, the field team visited sites throughout the health system, including the seven rural health centers in Boffa (Douprou, Mankountan, Tougnifily, Tamita, Koba, Colia, and Lisso), the Boffa prefecture hospital, the Boffa Prefectural Health Department (Direction Préfectoral de Santé), and the Division for the Prevention and Control of Disease (Division de la Prévention et la Lutte Contre les Maladies) within the Ministry of Health in Conakry (Fig 1). The eighth health center in Boffa, which is in the center of Boffa town, was closed because of staffing issues during the field visits and was not included in the study.

At each site the team used the questionnaires to interview a representative national public health system employee who was responsible for case assessment, disease reporting, and data analysis at his or her post. Specifically, the team interviewed the designated person in charge of disease surveillance at each health center and the prefecture hospital; the Medical Director of Disease Surveillance (le Médecin Chargé des Maladies) at the prefectural health department; and the Director of Epidemic Surveillance at the Division for the Prevention and Control of Disease at the Ministry of Health in Conakry. The case-based disease surveillance system in the Boffa prefecture hospital was still being established at the time of the interviews; therefore, results from the prefecture hospital interviews are not presented here.

Interview data were collected on paper and entered into a Microsoft Excel spreadsheet. Although a single individual served as the interviewee of record, multiple national public health system employees were frequently present during the interviews and contributed information. The results and conclusions presented are based primarily on review of case-based reporting documentation and the verbal information provided by interviewees.

## 3. Results

### 3.1 Overview of the Guinean health system, 2016

**Fig 2:**
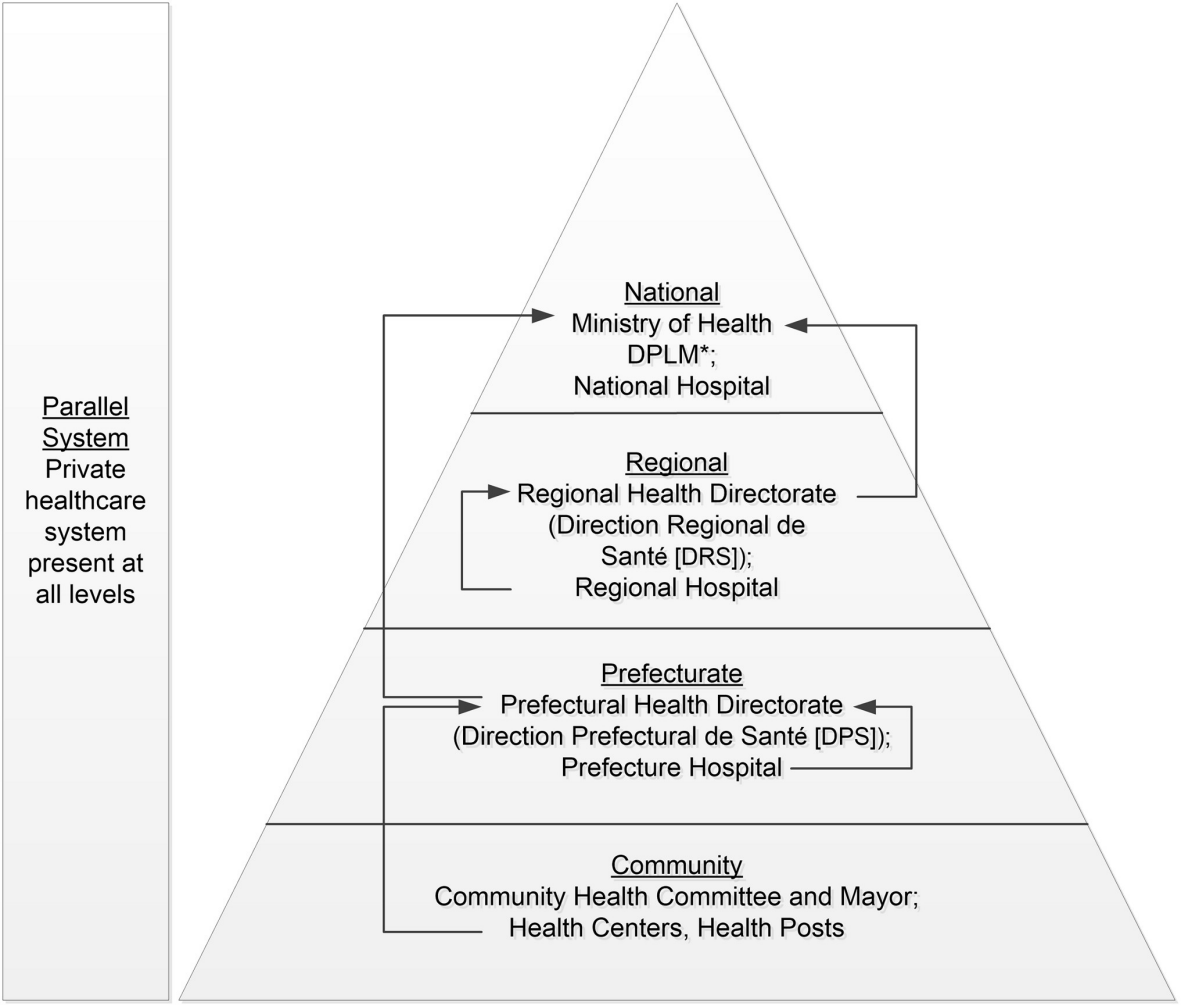
The Guinean health care system, 2016. (DPLM: Division de la Prévention et la Lutte Contre les Maladies, case-based reporting indicated by arrows).

To serve the population’s health care needs, each of Guinea’s 33 prefectures has government-operated clinical health care facilities, including health posts, health centers, and hospitals. The system is hierarchical with four levels: community, prefectural, regional, and national (Fig 2) [15]. Community-level health care is decentralized and provided at health posts (Postes de Santé) and health centers (Centres de Santé). Health centers, where most of the population accesses health care, provide basic medical care and treatment at the sub-prefecture level, including midwifery services, vaccinations, and essential medicines. In each prefecture, the prefectural health department oversees prefecture-level health policy implementation and provides support to the community level through the prefectural hospitals, prefectural offices for disease control and prevention, and administrative services. The regional health department oversees regional-level health policy implementation and provides support to the prefectural level through regional hospitals and administrative services. Nationally, the Ministry of Health is responsible for national health policy development, national health statistics, and oversight of health programs and health care delivery services; and provides support to the regional level through the Division for the Prevention and Control of Disease.

The case-based disease surveillance system in Guinea is embedded with the clinical health care system. Community health centers and the prefectural hospital report cases of notifiable diseases to the prefectural health department. At the time of this assessment, the prefectural health department submitted surveillance reports from the community and prefectural hospitals directly to the Ministry of Health at the national level, bypassing the regional-level health department (Direction Regional de Santé), which had primary responsibility for the national vaccine programs and was not involved in surveillance. At the national level, the Ministry of Health receives and analyzes surveillance data from all prefectures and oversees national disease prevention and control policy and implementation. Although a private health care system functions in parallel to the government-operated system at all levels, the private system is not under government authority for disease reporting (Fig 2).

### 3.2 Overview of case-based disease surveillance in Boffa, 2016

Per Guinea’s national disease surveillance strategy and as of September 2016, case-based disease surveillance in Boffa for priority diseases, including epidemic-prone diseases, began with case identification, typically at the health centers and prefecture hospital (Fig 2). These cases were reported by the health centers and prefecture hospital staff to the Boffa Prefectural Health Department, which subsequently reported cases to the Division for the Prevention and Control of Disease in Conakry. The regional health department was not part of this reporting structure. Ministry of Health protocol required two reporting mechanisms for case-based disease surveillance of epidemic-prone diseases: immediate and routine weekly reporting. Immediate reporting occurred when a health center or prefecture hospital care provider suspected or identified a case of an epidemic-prone disease and immediately notified the prefectural health department by telephone. The prefectural health department staff would then immediately notify the Ministry of Health by telephone. Routine weekly reporting occurred when each reporting level (community and prefectural) provided weekly aggregate case counts of the priority diseases, including epidemic-prone diseases, for the proceeding 7 days to the next level.

Partner organizations (e.g., WHO) operated parallel disease reporting systems in Guinea for weekly reporting of priority diseases, including malaria (Fig 2) [17]. Ministry of Health officials routinely engaged with representatives from these parallel systems to review case count data and reconcile discrepancies. Parallel reporting structures were not a focus of this study and are not discussed further.

#### 3.2.1 Community level: Health centers

*Organization*, *tools*, *and documentation*. The health center Manager oversaw both routine (i.e., case-based) disease surveillance and community-based surveillance activities, while the Deputy Manager served as a backup to the health center Manager for all disease surveillance activities. All health center Managers and Deputy Managers had been trained to function as community health worker supervisors. At all seven health centers (i.e., Douprou, Mankountan, Tougnifily, Tamita, Koba, Colia, and Lisso), the team observed that case definitions for case-based disease surveillance were available to staff to reference. Community-based surveillance case definitions were made available in April 2016 during community-based surveillance training activities. Five of the seven health centers (71%) recorded priority disease cases only in the general consultation register; interviewees at the remaining two (29%) reported that priority disease cases were also recorded in a designated case register. All health center staff maintained notebooks of weekly aggregate case counts of priority diseases, and interviewees indicated that staff spent time formatting notebooks of weekly aggregate case counts and, if applicable, case registers by hand. All health centers used the hand-formatted weekly case registers for routine weekly reporting of priority diseases by telephone to the prefectural health department. None of the health center interviewees reported routinely maintaining an outbreak log at their respective facility.

*Reporting*. During October 1, 2015–March 31, 2016, the health centers did not report to the prefectural health department any cases or outbreaks of the four epidemic-prone diseases included in this study (cholera, meningococcal meningitis, measles, and yellow fever). Therefore, immediate reporting of epidemic-prone diseases to the Boffa Prefectural Health Department and to the national Division for the Prevention and Control of Disease could not be assessed as part of this study.

Although interviewees at four health centers indicated that immediate reporting by phone to the prefectural health department is part of their protocol, health center staff do not routinely document this telephone call. Despite an absence of cases, the general consultation registers and notebooks of weekly aggregate case counts (indicating zero cases) appeared up to date at all the health centers. Interviewees at all seven health centers stated that routine weekly reporting to the prefectural health department had occurred during the period, although documentation of this reporting was not available for review.

*Feedback*. Six health centers (86%) reported that staff received confirmation of cases from the prefectural health department, by telephone (Table 1). However, all health centers requested that the prefectural health department provide written surveillance feedback (i.e., e-mailed reports). Two health centers said that their staff verbally provided surveillance information to the community at public meetings during October 1, 2015−March 31, 2016.

**Table 1:** Case-based disease surveillance feedback among seven* prefecture health centers, Boffa, Guinea, October 1, 2015–March 31, 2016.

Surveillance activities	Health Centers	
	N	%
Method of surveillance feedback that is/would be most helpful to health center		
Verbal report only	0	0
Written report only	6	86
Both written and verbal report	1	14
Health center provided surveillance information to their community		
Yes	2	29
No	5**	71
Method by which surveillance information was provided to community from health center		
Public meeting(s)/forum(s)	2	29
Community health worker meeting(s)/forums(s)	0	0
Print media	0	0
Radio	0	0
Other	0	0
Not applicable/No information provided	5	71

*Douprou, Mankountan, Tougnifily, Tamita, Koba, Colia, and Lisso.

** Of these five health centers, four had provided community feedback at public meetings previously but not during October 1, 2015–March 31, 2016.

*Data analysis*. Data analysis activities were minimal at health centers during October 1, 2015–March 31, 2016. One health center prepared summaries of case characteristics. Another health center mapped by hand the geographic distribution of cases prior to the period under study.

*Perceived needs*. Health center interviewees requested additional training on data management and analysis. They also requested that health centers be provided with preformatted case register books and rapid diagnostic tests. Other requests included improved provision of electricity because solar panels were not adequate to power equipment, information pamphlets on epidemic-prone diseases, and guidance on motivational strategies for community health workers.

#### 3.2.2 Prefecture level (Direction Préfectoral de la Santé)

*Organization*, *tools*, *and documentation*. The Medical Director of Disease Surveillance was the designated person in charge of disease surveillance at the prefectural health department. The team observed that case-based disease surveillance case definitions and community case definitions were available to the prefectural health department. Routine weekly reports of epidemic-prone diseases from the health centers were recorded in a registry at the prefectural health department.

*Reporting*. During October 1, 2015–March 31, 2016, the Boffa health centers did not report any cases or outbreaks of the four epidemic-prone diseases included in this study (cholera, meningococcal meningitis, measles, and yellow fever). Therefore, immediate reporting of epidemic-prone diseases to the next levels could not be assessed. However, the interviewee outlined the prefectural health department protocol for managing immediate reports as follows: (1) prefectural health department prepared a case investigation form and a laboratory test request form; (2) the laboratory sample and the investigation form were transported by a health department vehicle, although the transport vehicle frequently had no fuel because of a shortage of funds; and (3) the Medical Director of Disease Surveillance alerted the Ministry of Health by telephone, although these telephone calls were not typically documented.

The prefectural health department provided routine weekly reporting of epidemic-prone diseases to the Ministry of Health by telephone during October 1, 2015–March 31, 2016, per the interviewee. The interviewee also reported that the Ministry of Health provided feedback on routine weekly reports by telephone and in the form of quarterly written reports, although these written reports were not available for review. During the period under study, the interviewee indicated that all routine weekly reports from health centers were complete (i.e., contained case counts for all four epidemic-prone diseases) and received on time.

*Feedback*. The prefectural health department provided verbal reports of surveillance feedback to the health centers during October 1, 2015–March 31, 2016. All the health centers provided the prefectural health department with weekly reports of priority disease case numbers by telephone, although there were zero cases. The prefectural health department staff did not visit the health centers or hospital or request documentation to verify the reported data. The interviewee indicated that the prefectural health department preferred to receive all routine weekly reports of epidemic-prone diseases from the health centers and hospital via e-mail rather than by telephone.

*Data analysis*. During October 1, 2015–March 31, 2016, the Medical Director of Disease Surveillance used Microsoft Excel to prepare epidemiologic curves and used the EpiMap module in CDC’s EpiInfo to map the geographic distribution of cases. The prefectural health department staff did not prepare data summaries by case characteristics.

*Perceived needs*. Technical support and training in surveillance data management for prefectural health department staff involved in surveillance was requested. Other requests included provision of technical support and training in surveillance data management for staff at the health centers and the prefectural hospital. More frequent written surveillance feedback from the Ministry of Health was also preferred.

#### 3.2.3 National level: (Division de la Prévention et la Lutte Contre les Maladies)

*Organization*, *tools*, *and documentation*. The Division for the Prevention and Control of Disease used the IDSR framework to reference standard case definitions [6]. The staff maintained a hardcopy register of case data and entered case data into a Microsoft Excel file, which was saved to an onsite computer. Every 4 weeks a quality control check was conducted on the register and the electronic data; however, records of the quality control check were not available for review. Although the Division for the Prevention and Control of Disease had an organogram outlining the roles of all surveillance staff, it was under revision at the time of the site visit and was not available for review.

*Reportin*g. During October 1, 2015–March 31, 2016, the Boffa Prefectural Health Department reported zero cases of the four epidemic-prone diseases included in this study (cholera, meningococcal meningitis, measles, and yellow fever) to the national level. Therefore, immediate reporting of epidemic-prone diseases to the Division for the Prevention and Control of Disease could not be assessed. The Boffa Prefectural Health Department provided routine weekly reporting of epidemic-prone diseases to the national level by telephone during the period under study, although there were zero cases. However, the Division for the Prevention and Control of Disease would prefer for the Boffa Prefectural Health Department to provide these routinely weekly reports via e-mail.

*Feedback*. The Division for the Prevention and Control of Disease analyzed the case-based data received from the Boffa Prefectural Health Department and provided feedback by telephone; however, the content of the feedback was not specified. The Division for the Prevention and Control of Disease provided results of specific case investigations by telephone to the Boffa Prefectural Health Department, a practice informed by the experience of the EVD response. The interviewee indicated that during October 1, 2015–March 31, 2016, all weekly reports from Boffa of the four epidemic-prone diseases were complete (i.e., contained case counts for all four epidemic-prone diseases) and received on time. The completeness of the data received could not be verified because the Boffa Prefectural Health Department reported only aggregate case counts.

*Analysis*. During October 1, 2015–March 31, 2016, Division for the Prevention and Control of Disease staff, with supervision from the division surveillance director, used Microsoft Excel to prepare epidemiologic curves, create pivot tables of case-based data, and prepare summaries describing case characteristics. The staff also used WHO’s HealthMapper to map the geographic distribution of cases.

*Perceived needs*. To strengthen the regular review and analysis of weekly reported surveillance data, the interviewee requested harmonization and integration of case characteristics (i.e., age, sex, and vaccination status) and technical support and training on the surveillance data management capabilities of the DHIS2 [18].

## 4. Discussion

During October 1, 2015–March 31, 2016, in Boffa, Guinea, we observed zero reported cases of the four epidemic-prone diseases (cholera, meningococcal meningitis, measles, and yellow fever) in the routine weekly reporting from health care facilities to the prefectural health department and from the prefectural health department to the Division for the Prevention and Control of Disease. WHO data indicate three and two reported cases of cholera and yellow fever, respectively, across Guinea during a proximate time; therefore, it is feasible that there were no reported cases of these diseases in Boffa during the evaluation period [8, 10, 11, 16]. However, we expected to document reported cases of meningitis and measles through this surveillance system assessment, considering the country-wide case counts during a proximate time and the inclusion of the dry season (i.e., December through June when meningitis is most commonly identified) during our evaluation period [8, 10, 11, 16].

During the same time of year as our evaluation period, but 2 years later (October 2017−March 2018), DHIS2 data from the Guinean national health information system (Système National d’Information Sanitaire) also indicate no reported cases of the four epidemic-prone diseases in Boffa. However, these DHIS2 data for October 2017−March 2018 do indicate reported cases among all four neighboring prefectures (Boké, Dubréka, Fria, and Télimélé) as follows: yellow fever (75 cases), meningitis (9 cases), measles (50 cases), and cholera (0 cases). Considering the similarities of Boffa to these neighboring prefectures, these findings suggest possible low sensitivity in the Boffa case-based surveillance system, not only during the evaluation time period but also during October 2017−March 2018. Further, the presence of EVD in the region during the evaluation time period, leading to a redirection of public health activities and change in care-seeking behaviors, could be associated with this low sensitivity [19]. Opportunities to strengthen surveillance and prevent future disease outbreaks may be informed by further exploration of this low sensitivity (e.g., assessing the knowledge, attitudes, and practices of clinic staff in identifying epidemic-prone diseases).

To our knowledge, this is the first published documentation of the health care and public health structure as it relates to the case-based disease surveillance system in a rural Guinean prefecture during the era of the largest EVD outbreak in history. The case-based disease surveillance system in Boffa, Guinea during October 1, 2015–March 31, 2016, was simple in design, with three levels of case reporting and data exchange by telephone (community to prefectural) and by e-mail (prefectural to national). Despite zero reported cases, routine weekly reporting of (zero) cases from health care facilities to the prefectural health department and from the prefectural health department to the national level appeared to be complete and were received on time, as reported by the interviewees. However, the system relied heavily on telephone reporting with minimal documentation, making it impossible to fully assess data quality. Notably, interviewees at all levels of the disease-reporting structure requested additional training on data management and analysis. Other requests of support from the health centers included guidance on motivational strategies for community health workers, provision of rapid diagnostic tests, and information pamphlets on epidemic-prone diseases for the community. Based on this assessment, we conclude that system simplicity could be improved with the provision of weekly case count notebooks and case registers that are preformatted according to data collection forms in use country-wide. Preformatted tools would reduce the amount of staff time use to develop notebooks and would ensure that case data can be efficiently accessed for data analyses. Partners should consider capacity building throughout the system for data analysis, including developing epidemiologic curves, mapping the geographic distribution of cases, and data summaries by case characteristics. Any surveillance data analysis training should encompass interpretation, feedback to stakeholders and data providers, and use of results to inform public health action.

Additional description of the verbal feedback provided by the prefectural health department to health centers (e.g., case confirmation, data quality) could inform development of methods and indicators for data quality assessments and validation of weekly reports. In collaboration with the Division for Control and Prevention of Disease and representative prefectural health departments, partners could help determine what enhancements can be made to the quarterly report format for improved usefulness and quality. Further description of the data analyses being conducted at the prefectural and national levels could inform understanding of the ideal data analysis responsibilities at each level. This information could direct workforce training for data management, quality control, and use of surveillance data analysis results. Partners may also consider developing DHIS2 surveillance sub-system training modules and supplemental training on data interpretation and analysis tools (e.g., case mapping, data harmonization) and evaluate the impact of this training on the quality of data analysis and reporting at prefectural levels.

Our evaluation findings are consistent with similar case-based disease surveillance system evaluations in Africa [20–22]. Systems are simple with reporting initiated from the health system level and combined centrally [22]. A need for additional training in data analysis and strengthening of surveillance feedback has been documented in similar evaluations [20–22]. For example, the authors of an evaluation of Guinea-Bissau’s cholera surveillance system noted that continued training on surveillance, data analysis, and outbreak response was needed to improve the sensitivity of the system. When evaluating the IDSR for infectious disease control in northern Ghana, researchers identified through key informant interviews that a lack of central-level feedback (e.g., more real-time feedback on data discrepancies) from district health administration to peripheral health workers presented challenges to consistency of reporting and overall surveillance system functioning, a finding consistent with our data from this evaluation [20]. An evaluation of measles surveillance in Nigeria also noted that, as with Guinea, the surveillance system faced a level of instability based on its reliance on donors for both funding and technical support. The study authors went on to propose that the country move to take complete ownership of the case-based surveillance system to ensure the system’s sustainability through provision of needed funding and logistical support [21].

This assessment had several limitations. The results and conclusions presented are based primarily on the verbal information provided by interviewees and supplemented with our assessments of the case log books. Although the field team made every effort to confirm interviewee statements, limited documentation was available to further validate interview results. Without reported cases, immediate reporting at each level of the system could not be assessed. We used U.S. CDC guidelines for surveillance system evaluations, with a focus on the surveillance system’s operations, resources, and attributes (i.e., simplicity, data quality) [14]. However, there are additional elements of a surveillance system evaluation that could be considered for future work, including assessment of other system attributes (e.g., sensitivity, positive predictive value, representativeness). Finally, our results of this assessment in Boffa are not necessarily generalizable to other prefectures in Guinea.

Through this evaluation we did not determine whether the absence of reported cases, specifically of meningitis and measles, in Boffa is a true reflection of the burden of these diseases. However, these findings suggest the possibility of low sensitivity of the surveillance system, compared to DHIS2 case counts, as described above. Considering this possibility, technical assistance partners working in the region could conduct future surveillance system–strengthening activities around two focus areas—surveillance data quality and data analysis—which align with the GHSA Action Packages of Real-time Surveillance and Workforce Development [5]. These Action Packages, which provide focus and structure to priority technical areas, promote strong case-based surveillance and community-based surveillance and a workforce trained to meet relevant epidemiologic core competencies in compliance with IHR [4].

In conclusion, we established a baseline understanding of the case-based surveillance system to inform our community-based surveillance implementation activities in Boffa in the aftermath of the largest EVD outbreak in history [1]. This surveillance system evaluation provides a framework for understanding the flow of information from the community to the national level in Guinea immediately following the outbreak. Results could be used to inform development of regionally specific Global Health Security surveillance indicators to measure and evaluate the effect of project interventions over time and to adjust program interventions, as needed, while supporting the government of Guinea in achieving its GHSA goals.

## Supporting information

S1 File Data collection tools (English). (PDF)

S2 File Data collection tools (French). (PDF)

## References

[1] Centers for Disease Control and Prevention. 2014–2016 Ebola outbreak in West Africa [Internet]—Summary. 2017 [cited June 15 2018]. Atlanta, GA: Centers for Disease Control and Prevention https://www.cdc.gov/vhf/ebola/outbreaks/2014-west-africa/.

[2] Baize S, Pannetier D, Oestereich L, Rieger T, Koivogui L, Magassouba N, et al Emergence of Zaire Ebola virus disease in Guinea. N Engl J Med. 2014 10 9;371(15): 1418–1425. 10.1056/NEJMoa1404505 24738640

[3] Kruk ME, Myers M, Varpilah ST, Dahn BT. What is a resilient health system? Lessons from Ebola. Lancet. 2015 5 9;385(9980): 1910–1912. 10.1016/S0140-6736(15)60755-3 25987159

[4] World Health Organization. International Health Regulations, 2nd ed 2005 [cited June 15 2018]. Geneva: World Health Organization http://whqlibdoc.who.int/publications/2008/9789241580410_eng.pdf.

[5] Global Health Security Agenda. Implementing the Global Health Security Agenda: Progress and Impact from U.S. Government Investments. 2018 [cited June 15 2018]. https://www.ghsagenda.org/docs/default-source/default-document-library/global-health-security-agenda-2017-progress-and-impact-from-u-s-investments.pdf?sfvrsn=4

[6] Centers for Disease Control and Prevention. Global disease detection operations center: event-based surveillance. 2016 [cited June 15 2018]. Atlanta, GA: Centers for Disease Control and Prevention http://www.cdc.gov/globalhealth/healthprotection/gddopscenter/how.html.

[7] World Health Organization. Early detection, assessment and response to acute public health events: implementation of early warning and response with a focus on event-based surveillance. 2014 [cited June 15 2018]. http://apps.who.int/iris/bitstream/handle/10665/112667/WHO_HSE_GCR_LYO_2014.4_eng.pdf;jsessionid=531F507A20CED289E033D9D315354DA9?sequence=1.

[8] Guinean Ministry of Health. Guide Technique pour La surveillance Integree De La Maladie Et La Riposte En Guinee. 2011 December.

[9] Guinean Ministry of Health. Plan de Relance du Systeme de Sante (2015–2017). 2015 April [cited June 15 2018]. http://www.invest.gov.gn/document/plan-relance-systeme-sante-guinee-2015-2017.

[10] World Health Organization. Technical guidance for integrated disease surveillance and response for the African region, 2nd edition. Geneva: 2010

[11] World Health Organization. Global health observatory data repository–cholera number of reported cases. no date [cited June 15 2018]. http://apps.who.int/gho/data/node.main.175?lang=en.

[12] Rebaudet S, Mengel MA, Koivogui L, Moore S, Mutreja A, Kande Y, et al Deciphering the origin of the 2012 cholera epidemic in Guinea by integrating epidemiological and molecular analyses. PLoS Negl Trop Dis. 2014 6;8(6): e2898 10.1371/journal.pntd.0002898 24901522 PMC4046952

[13] City Population. BOFFA Prefecture in Guinea. 2019 [cited October 22 Oldenburg: Thomas Brinkhoff. https://www.citypopulation.de/en/guinea/admin/11__boffa/.

[14] German RR, Lee LM, Horan JM, Milstein RL, Pertowski CA, Waller MN, et al Updated guidelines for evaluating public health surveillance systems: recommendations from the Guidelines Working Group. MMWR Recomm Rep. 2001 7 27;50(RR-13): 1–35; quiz CE31-37. 18634202

[15] Ministere de la Sante et de l’Hygiene Publique, Direction Nationale des Etablissements Hospitaliers et de Soins. Organization de system de sante Guineen. 2014

[16] World Health Organization. WHO vaccine-preventable diseases: monitoring system 2016 global summary. 2018 [cited June 15 2018]. World Health Organization http://apps.who.int/immunization_monitoring/globalsummary/countries?countrycriteria%5Bcountry%5D%5B%5D=GIN&commit=OK.

[17] World Health Organization. World malaria report. 2018 [cited October 22 2019]. Geneva, Switzerland: World Health Organization. https://apps.who.int/iris/bitstream/handle/10665/275867/9789241565653-eng.pdf?ua=1.

[18] District Health Information System Version 2 (DHIS 2). DHIS 2.29 is here. no date [cited June 15 2018]. https://www.dhis2.org/.

[19] Centers for Disease Control and Prevention. 2014–2016 Ebola outbreak in West Africa [Internet]. 2017 [cited January 27 2020]. Atlanta, GA: Centers for Disease Control and Prevention https://www.cdc.gov/vhf/ebola/history/2014-2016-outbreak/distribution-map.html.

[20] Adokiya MN, Awoonor-Williams JK, Barau IY, Beiersmann C, Mueller O. Evaluation of the integrated disease surveillance and response system for infectious diseases control in northern Ghana. BMC Public Health. 2015 2 4;15: 75 10.1186/s12889-015-1397-y 25648630 PMC4331174

[21] Ameh CA, Sufiyan MB, Jacob M, Waziri NE, Olayinka AT. Evaluation of the Measles Surveillance System in Kaduna State, Nigeria (2010–2012). Online J Public Health Inform. 2016;8(3): e206 10.5210/ojphi.v8i3.7089 28210427 PMC5302462

[22] Sanchez-Padilla E. Evaluation of the cholera surveillance system in Guinea Bissau, Report v. 4.4. 2009, December [cited June 15 2018]. https://www.unicef.org/evaldatabase/files/GBIS-2010-001-1.pdf.

