# Supplementary material for: Surveillance system assessment in Guinea: Training needed to strengthen data quality and analysis, 2016
Source: PLoS One. 2020 Jun 25;15(6):e0234796. doi: 10.1371/journal.pone.0234796 (PMC7316275; doi:10.1371/journal.pone.0234796)
Supplement: S1 File — (PDF) [file pone.0234796.s001.pdf]

Disease surveillance in Boffa, Guinea — Data collection tool: Health center

Health Center Name: ________________________________

Interviewer's Name: ________________________________ **Date:**________________________________

This tool will be used collect baseline information from health centers in Boffa, Guinea about public health surveillance, including indicator case-based and community-based surveillance. Although the focus is on surveillance for 4 epidemic-prone diseases (i.e., cholera, measles, meningitis, and yellow fever) during the time period of October 1, 2015–March 1, 2016, several general surveillance questions are also included in the tool. The data collected will inform Epi-Detecte strategies to effectively improve disease surveillance in Guinea and serve as a baseline for determining the impact of planned Epi-Detecte interventions on basic surveillance system functions.

Interviewer Guidance:

- The interviewer should contact the health center in advance to arrange a site visit with the designated person in charge of disease surveillance at the health center (i.e., head of disease surveillance). If there is no designated person in this role, the health center's medical director can serve in this capacity.
- At the beginning of the site visit, the interviewer should explain to the head of disease surveillance and to any other persons assisting with data collection (i.e., the interviewee(s)) the purpose of this activity and how the data will be used (see paragraph above). The interviewer should assure the interviewee(s) that they will not be personally identified in the results.
- During the site visit, the interviewer should complete each question in this data collection tool by interviewing the head of disease surveillance and asking for his/her assistance in collecting the requested information and materials. The interviewer should attempt to complete the tool in a single site visit. However, if needed, a follow-up visit could be arranged within 1 week of the initial visit to complete data collection.
- The interviewer should use the "Checklist of materials" at the bottom of this page to ensure that, in addition to completing the questions, all other requested and available materials are obtained.
- Additional interviewer guidance and instruction can be found throughout the document in italics.
- Note that throughout the document, meningococcal meningitis is referred to as meningitis.

Acronyms and Definitions:

DPS = Prefectural Health Directorate (district level)
IDSR = Integrated Disease Surveillance and Response
MOH = Ministry of Health
Priority diseases = African public health priorities, as specified by the IDSR framework and MOHs; diseases which are largely preventable with well-known and efficacious responses
Epidemic-prone diseases = Subset of priority diseases with a high epidemic potential to cause serious public health impact due to their ability to spread rapidly internationally
Outbreak = Occurrence of cases of disease in excess of what would normally be expected in a defined community, geographic area, or season

Checklist of materials for interviewer to obtain (either copy or take pictures), if available:

☐ Community-based surveillance case definitions for priority diseases (question 3)
☐ Standard case definitions for priority diseases (question 4)
☐ Surveillance feedback from DPS to the health center (question 11)
☐ Surveillance information from the health center to the community (question 14)
☐ Data analyses for priority diseases (question 15)

Please ask the interviewee(s) the following questions and request assistance in collecting the information, as indicated. If needed, use the "Comments" space after each question to describe additional relevant detail and/or challenges in obtaining the information.

Background

1. What is the title and role of the designated person in charge of disease surveillance at the health center (i.e., head of disease surveillance)? ____
   ____

Community-based Disease Surveillance

2. What is the title and role of the designated person in charge of community-based surveillance in association with the health center?
   ____
   ____

3. *Ask to see the community-based surveillance case definitions for priority diseases (including epidemic-prone diseases), and, if available, request copies and/or take pictures of these community-based surveillance case definitions.*
   Are community-based surveillance case definitions for priority diseases (including epidemic-prone diseases) available at the health center for staff to reference? *(Check only one)*
   ☐ Yes
   ☐ No
   ☐ Not sure *(explain in comments)*
   Comments: ____

Health Center-based Disease Surveillance

4. *Ask to see the standard case definitions for priority diseases (including epidemic-prone diseases) that are used by the health center staff and, if available, request copies and/or take pictures of these standard case definitions*.

   Are standard case definitions for priority diseases (including epidemic-prone diseases) available at the health center for staff to reference? *(Check only one)*

   ☐ Yes
   ☐ No
   ☐ Not sure *(explain in comments)*

   Comments: ____

5. *Ask to see the health center's case registry where diagnoses of priority diseases (including epidemic-prone diseases) are recorded.*

   Are diagnoses of priority diseases (including epidemic-prone diseases) recorded in a case registry at the health center according to a standard case definition? *(Check only one)*

   ☐ Yes
   ☐ No
   ☐ Not sure *(explain in comments)*

   Comments: ____

6. *Review the entries in the case registry, if available.*

   Does the health center's case registry appear to be up to date? *(Check only one)*

   ☐ Not applicable - there is no case registry
   ☐ Yes
   ☐ No
   ☐ Not sure *(explain in comments)*

   Comments: ____

   ____

7. *In consultation with the interviewee(s) and using the health center's case registry and/or other available documentation, complete the following health center case count table of 4 epidemic-prone diseases for the time period **October 1, 2015–March 31, 2016**.*

**Disease**	**Number of Cases**
Cholera	
Measles	
Meningitis	
Yellow fever	

Reporting and Feedback

8. *Ask if the health center documents the required immediate reporting of epidemic-prone diseases to DPS, and, if yes, ask to see this documentation.*

   Did the health center document the required immediate reporting of epidemic-prone diseases to DPS during **October 1, 2015–March 31, 2016**? *(Check only one)*

   ☐ Yes, there is documentation that all cases of epidemic-prone diseases were immediately reported to DPS during the time period. (*Describe method of documentation*: ____)

   ☐ Yes, there is documentation that some cases of epidemic-prone diseases were immediately reported to DPS during the time period. (*Describe method of documentation*: ____)

   ☐ No, there is no documentation indicating that cases of epidemic-prone diseases were immediately reported to DPS during the time period.

   ☐ Not sure *(explain in comments)*

Comments: ____

9. *Ask if the health center documents their routine* *<u>weekly reporting</u>* *of epidemic-prone diseases to DPS, and, if yes, ask to see this documentation.*
   Did the health center document their routine <u>weekly reporting</u> of epidemic-prone diseases to DPS during **<u>October 1, 2015–March 31, 2016</u>**? *(Check only one)*
   ☐ Yes, there is documentation that <u>all</u> weekly reports of epidemic-prone diseases were provided to DPS during the time period. (*Describe method of documentation*: ______________________________)
   ☐ Yes, there is documentation that <u>some</u> weekly reports of epidemic-prone diseases were provided to DPS during the time period (*Describe method of documentation*: ______________________________)
   ☐ No, there is no documentation indicating that weekly reports of epidemic-prone diseases were provided to DPS during the time period.
   ☐ Not sure *(explain in comments)*

Comments: ______________________________

10. Did the health center receive any surveillance feedback (e.g., phone call acknowledging receipt of report, information on data quality, data summary) from DPS during **<u>October 1, 2015–March 31, 2016</u>**? *(Check only one)*
    ☐ Yes
    ☐ No
    ☐ Not sure *(explain in comments)*

Comments: ______________________________

11. How did DPS provide surveillance feedback to the health center during **<u>October 1, 2015–March 31, 2016</u>**?
    (Check all that apply and <u>request copies and/or take pictures, if available</u>)
    _____Not applicable – no surveillance feedback was provided
    _____Written report
    _____Verbal report
    _____Other (*please describe*: ______________________________)

12. What surveillance feedback from DPS is most helpful or would be most helpful to the health center? *(Please describe)*
    ____
    ____
    ____
    ____

13. Did the health center provide any surveillance information (e.g., data summary, public health alerts or notifications) to the community during **October 1, 2015–March 31, 2016**? *(Check only one)*

☐ Yes
☐ No
☐ Not sure *(explain in comments)*

Comments: ____

14. How did the health center provide surveillance information to the community during **October 1, 2015–March 31, 2016**? *(Check all that apply and request copies and/or take pictures, if available)*

_____Not applicable – no surveillance information was provided
_____Public meeting(s) or forum(s)
_____Community health worker meeting(s) or forums(s)
_____Print media
_____Radio
_____Other (*please describe*: ____)

<u>Analysis</u>

15. *Ask to review analyses of health center surveillance data for priority diseases*. In consultation with the interviewee(s), complete the following table to characterize the analysis activities at the health center during **<u>October 1, 2015–March 31, 2016</u>**, and <u>request copies and/or take pictures of analyses, if available</u>***.**

**Activity**	**Answer** *(circle one per question)*	**Position/Title of the Person Who Completed the Analysis**	**What tools** were used to complete the analysis?**	**Comments**
Plotted the numbers of cases on a graph	Yes No			
Plotted the distribution of cases on a map	Yes No			
Prepared summaries describing cases by characteristics (e.g., age, sex, education level, vaccination status, occupation or place of employment)	Yes No			

*Analyses for any priority disease, including but not limited to epidemic-prone diseases

**Examples: pen and paper, calculator, computer

Comments: ____
____

16. In what way(s) could Epi-Detecte best help to strengthen the regular review and analysis of weekly-reported surveillance data?
____
____
____
____
____
____

Disease Outbreaks

17. *Ask to see the log where suspected disease outbreaks are recorded.*
    Are suspected disease outbreaks recorded in a log at the health center? *(Check only one)*
    ☐ Yes
    ☐ No
    ☐ Not sure *(explain in comments)*

Comments: ______________________________

18. *Review the entries in the outbreak log.*
    Does the disease outbreak log appear to be to date? *(Check only one)*
    ☐ Not applicable – there is no outbreak log
    ☐ Yes
    ☐ No
    ☐ Not sure *(explain in comments)*

Comments: ______________________________

19. *In consultation with the interviewee(s) and using the outbreak log and/or other available documentation, complete the following health center outbreak count table of the 4 epidemic-prone diseases for the time period **October 1, 2015–March 31, 2016**.*

**Disease**	**Number of Outbreaks Identified**	**Number of Outbreaks Investigated**	**Number of Outbreaks Reported to DPS**	**List the response resources, if any, that were provided by MOH for outbreak investigation**
Cholera				
Measles				
Meningitis				
Yellow fever				

Comments: ______________________________

Please provide any other comments below.

End of data collection tool

Disease surveillance in Boffa, Guinea — Data collection tool: DPS

Interviewer's Name: ___________________________ **Date:**_____________________________

This tool will be used collect baseline information from the Prefectural Health Directorate (DPS) in Boffa, Guinea about public health surveillance, including indicator case-based and community-based surveillance. Although the focus is on surveillance for 4 epidemic-prone diseases (i.e., cholera, measles, meningitis, and yellow fever) during the time period October 1, 2015–March 1, 2016, several general surveillance questions are also included in the tool. The data collected will inform Epi-Detecte strategies to effectively improve disease surveillance in Guinea and serve as a baseline for determining the impact of planned Epi-Detecte interventions on basic surveillance system functions.

Interviewer Guidance:

- The interviewer should contact the DPS in advance to arrange a site visit with the Medicine Charge de Maladies (MCM). If this is not possible, the interviewer should arrange a site visit with an official at the DPS who is knowledgeable about disease surveillance.
- At the beginning of the site visit, the interviewer should explain to the MCM or other official and to any other persons assisting with data collection (i.e., the interviewee(s)) the purpose of this activity and how the data will be used (see paragraph above). The interviewer should assure the interviewee(s) that they will not be personally identified in the results.
- During the site visit, the interviewer should complete each question in this data collection tool by interviewing the MCM and asking for his/her assistance in collecting the requested information and materials. The interviewer should attempt to complete the tool in a single site visit. However, if needed, a follow-up visit could be arranged within 1 week of the initial visit to complete data collection.
- The interviewer should use the "Checklist of materials" at the bottom of this page to ensure that, in addition to completing the questions, all other requested and available materials are obtained.
- Additional interviewer guidance and instruction can be found throughout the document in italics.
- Note that throughout the document, meningococcal meningitis is referred to as meningitis.

Acronyms and Definitions:

DPLM = Division of the Prevention and Control of Disease
DPS = Prefectural Health Directorate (district level)
IDSR = Integrated Disease Surveillance and Response
MCM = Medicine Charge de Maladies
MOH = Ministry of Health
Priority diseases = African public health priorities, as specified by the IDSR framework and MOHs; diseases which are largely preventable with well-known and efficacious responses
Epidemic-prone diseases = Subset of priority diseases with a high epidemic potential to cause serious public health impact due to their ability to spread rapidly internationally
Outbreak = Occurrence of cases of disease in excess of what would normally be expected in a defined community, geographic area, or season

Checklist of materials for interviewer to obtain (either copy or take pictures), if available:

☐ Community-based surveillance case definitions for priority diseases (question 2)
☐ Standard case definitions for priority diseases (question 3)
☐ Surveillance feedback from DPLM to DPS (question 12)
☐ Surveillance feedback from DPS to Boffa health centers or prefectural hospital (question 15)
☐ Data analyses for priority diseases (question 16)

Please ask the interviewee(s) the following questions and request assistance in collecting the information, as indicated. If needed, use the "Comments" space after each question to describe additional relevant detail and/or challenges in obtaining the information.

Community-based Disease Surveillance

1. What is the title and role of the designated person in charge of community-based surveillance in association with the DPS?
   (If there is no one at the DPS who serves in this function, please indicate "Not applicable" in the space below.)
   ____
   ____

2. *Ask to see the community-based surveillance case definitions for priority diseases (including epidemic-prone diseases), and, if available, request copies and/or take picture(s) of these community-based surveillance case definitions.*
   Are community-based surveillance case definitions for priority diseases (including epidemic-prone diseases) available at the DPS for staff to reference? *(Check only one)*
   ☐ Yes
   ☐ No
   ☐ Not sure *(explain in comments)*
   Comments: ____

General Surveillance and Reporting

3. *Ask to see the standard case definitions for priority diseases (including epidemic-prone diseases) that are used by the DPS staff and, if available, request copies and/or take pictures of these standard case definitions.*
   Are standard case definitions for priority diseases (including epidemic-prone diseases) available at DPS for staff to reference?
   (Check only one)
   ☐ Yes
   ☐ No
   ☐ Not sure (*explain in comments*)
   Comments: ____

4. *Ask to see the registry where DPS records the routine <u>weekly reporting</u> of epidemic-prone diseases from the Boffa health centers and prefectural hospital.*
   Are the routine <u>weekly reports</u> of epidemic-prone diseases from the Boffa health centers and prefectural hospital recorded in a registry at DPS?
   (Check only one)
   ☐ Yes
   ☐ No
   ☐ Not sure *(explain in comments)*
   Comments: ____

5. *In consultation with the interviewee(s) and using the registry and/or other available documentation, complete the following tables of routine <u>weekly reporting</u> by month of epidemic-prone diseases to the DPS from the Boffa health centers and prefectural hospital for the time period **<u>October 1, 2015–March 31, 2016.</u>** (See the following 2 pages for the tables)*
   For each week, determine:
   1) If the weekly report from the facility was received at all ("Received")
   If YES, place an "X" in the designated box.
   If NO, place a "0" in the designated box.
   2) If the weekly report from the facility was received on-time ("On-time")
   If YES, place an "X" in the designated box.
   If NO, place a "0" in the designated box.

   **Note that a 5th week is included for each month but may not be needed, depending on the reporting schedule. If not needed, leave the 5th week box(es) blank.*

Month	Week of Month	Report Status	Reporting Facility								
			Colia	Mankountan	Koba	Lisso	Tamita	Douprou	Tougnify	Boffe Centre	Prefectural Hospital
October 2015	1	Received									
		On-time									
	2	Received									
		On-time									
	3	Received									
		On-time									
	4	Received									
		On-time									
	5*	Received									
		On-time									
November 2015	1	Received									
		On-time									
	2	Received									
		On-time									
	3	Received									
		On-time									
	4	Received									
		On-time									
	5*	Received									
		On-time									
December 2015	1	Received									
		On-time									
	2	Received									
		On-time									
	3	Received									
		On-time									
	4	Received									
		On-time									
	5*	Received									
		On-time									

Month	Week of Month	Report Status	Reporting Facility								
			Colia	Mankountan	Koba	Lisso	Tamita	Douprou	Tougnify	Boffe Centre	Prefectural Hospital
January 2016	1	Received									
		On-time									
	2	Received									
		On-time									
	3	Received									
		On-time									
	4	Received									
		On-time									
	5*	Received									
		On-time									
February 2016	1	Received									
		On-time									
	2	Received									
		On-time									
	3	Received									
		On-time									
	4	Received									
		On-time									
	5*	Received									
		On-time									
March 2016	1	Received									
		On-time									
	2	Received									
		On-time									
	3	Received									
		On-time									
	4	Received									
		On-time									
	5*	Received									
		On-time									

6. *In consultation with the interviewee(s) and using the registry and/or other available documentation, determine the method(s) by which the routine* *__weekly reports__* *were received by DPS from each of the reporting facilities during the time period* ***__October 1, 2015–March 31, 2016.__***
For each reporting facility, place an "X" in the appropriate box(es), indicating all methods that apply.

**Method of Report to DPS**	**Reporting Facility**								
	**Colia**	**Mankountan**	**Koba**	**Lisso**	**Tamita**	**Douprou**	**Tougnify**	**Boffe Centre**	**Prefectural Hospital**
Telephone									
Fax									
Text									
Email									
Other (describe)									

7. What method of weekly reporting from facilities is most helpful or would be most helpful to the DPS? *(Please describe)*

____
____
____
____

8. *In consultation with the interviewee(s) and using the registry and/or other available documentation, complete the following case count tables by Boffa health center and prefectural hospital of 4 epidemic-prone diseases for the time period* ***October 1, 2015–March 31, 2016****.*

**Reporting Facility and Disease**	**Number of Reported Cases***	**Number of Reported Cases* with Confirmatory Laboratory Testing *Performed***	**Number of Reported Cases with Confirmed Laboratory Test *Results***	**Total Number of Reported Cases* Investigated by DPS**	**Total Number of Reported Cases* With a Completed Case Investigation Form**
**Colia**					
Cholera					
Measles					
Meningitis					
Yellow fever					
**Mankountan**					
Cholera					
Measles					
Meningitis					
Yellow fever					
**Koba**					
Cholera					
Measles					
Meningitis					
Yellow fever					

*Total number of cases, including confirmed and suspected cases

Comments: ____

**Reporting Facility and Disease**	**Number of Reported Cases***	**Number of Reported Cases* with Confirmatory Laboratory Testing *Performed***	**Number of Reported Cases with Confirmed Laboratory Test *Results***	**Total Number of Reported Cases* Investigated by DPS**	**Total Number of Reported Cases* With a Completed Case Investigation Form**
**Lisso**					
Cholera					
Measles					
Meningitis					
Yellow fever					
**Tamita**					
Cholera					
Measles					
Meningitis					
Yellow fever					
**Douprou**					
Cholera					
Measles					
Meningitis					
Yellow fever					

*Total number of cases, including confirmed and suspected cases

Comments: ____

Reporting Facility and Disease	Number of Reported Cases*	Number of Reported Cases* with Confirmatory Laboratory Testing *Performed*	Number of Reported Cases with Confirmed Laboratory Test *Results*	Total Number of Reported Cases* Investigated by DPS	Total Number of Reported Cases* With a Completed Case Investigation Form
**Tougnify**					
Cholera					
Measles					
Meningitis					
Yellow fever					
**Boffe Centre**					
Cholera					
Measles					
Meningitis					
Yellow fever					
**Prefectural hospital**					
Cholera					
Measles					
Meningitis					
Yellow fever					

*Total number of cases, including confirmed and suspected cases

Comments: ____

____

9. *Ask if the DPS documents the required immediate reporting of epidemic-prone diseases to DPLM, and, if yes, ask to see this documentation.*
   Did the DPS document the required immediate reporting of epidemic-prone diseases to DPLM during **October 1, 2015–March 31, 2016**? *(Check only one)*
   ☐ Yes, there is documentation that all cases of epidemic-prone diseases were immediately reported to DPLM during the time period.
   (*Describe method of documentation*: ______________________________)
   ☐ Yes, there is documentation that some cases of epidemic-prone diseases were immediately reported to DPLM during the time period.
   (*Describe method of documentation*: ______________________________)
   ☐ No, there is no documentation indicating that cases of epidemic-prone diseases were immediately reported to DPLM during the time period.
   ☐ Not sure *(explain in comments)*

   Comments: ______________________________

10. *Ask if the DPS documents their routine weekly reporting of epidemic-prone diseases to DPLM, and, if yes, ask to see this documentation.*
   Did the DPS document the routine weekly reporting of epidemic-prone diseases to DPLM during **October 1, 2015–March 31, 2016**? *(Check only one)*
   ☐ Yes, there is documentation that all weekly reports of epidemic-prone diseases were provided to DPLM during the time period.
   (*Describe method of documentation*: ______________________________)
   ☐ Yes, there is documentation that some weekly reports of epidemic-prone diseases were provided to DPLM during the time period
   (*Describe method of documentation*: ______________________________)
   ☐ No, there is no documentation indicating that weekly reports of epidemic-prone diseases were provided to DPLM during the time period.
   ☐ Not sure *(explain in comments)*

   Comments: ______________________________

Surveillance Feedback

11. Did the DPS receive any surveillance feedback (e.g., phone call acknowledging receipt of report, information on data quality, data summary) from DPLM during **October 1, 2015–March 31, 2016**?
   (Check only one)
   ☐ Yes
   ☐ No
   ☐ Not sure *(explain in comments)*

   Comments: ______________________________

12. How did DPLM provide surveillance feedback to DPS during **October 1, 2015–March 31, 2016**?
(Check all that apply and request copies and/or take pictures, if available)
_____Not applicable – no surveillance feedback was provided
_____Written report
_____Verbal report
_____Other (*please describe*: ____)

13. What surveillance feedback from DPLM is most helpful or would be most helpful to the DPS? *(Please describe)*
____
____
____
____

14. Did the DPS provide any surveillance feedback (e.g., phone call acknowledging receipt of report, information on data quality, data summary) to the Boffa health centers or prefectural hospital during **October 1, 2015–March 31, 2016**?
(Check only one)
☐ Yes
☐ No
☐ Not sure *(explain in comments)*

Comments: ____

15. How did the DPS provide surveillance feedback to the Boffa health centers or prefectural hospital during **October 1, 2015–March 31, 2016**?
(Check all that apply and request copies and/or take pictures, if available)
_____Not applicable – no surveillance feedback was provided
_____Written report
_____Verbal report
_____Other (*please describe*: ____)

Analysis

16. *Ask to review analyses of DPS surveillance data for priority diseases*. In consultation with the interviewee(s), complete the following table to characterize the analysis activities at the DPS during* ***October 1, 2015–March 31, 2016****, and request copies of analyses and/or take pictures, if available.*

**Activity**	**Answer** *(circle one per question)*	**Position/Title of the Person Who Completed the Analysis**	**What tools** were used to complete the analysis?**	**Comments**
Plotted the numbers of cases on a graph	Yes No			
Plotted the distribution of cases on a map	Yes No			
Prepared summaries describing cases by characteristics (e.g., age, sex, education level, vaccination status, occupation or place of employment)	Yes No			

*Analyses for any priority disease, including but not limited to epidemic-prone diseases

**Examples: pen and paper, calculator, computer

Comments: ____

17. In what way(s) could Epi-Detecte best help to strengthen the regular review and analysis of weekly-reported surveillance data?

____
____
____
____
____
____

Please provide any other comments below.

End of data collection tool

Disease surveillance in Boffa, Guinea — Data collection tool: DPLM

Interviewer's Name: ________________________ **Date:**__________________________

This tool will be used collect baseline information from the Division of the Prevention and Control of Disease (DPLM) in Guinea about public health surveillance, including indicator case-based and community-based surveillance during October 1, 2015–March 1, 2016 in Boffa, Guinea. Although the focus is on surveillance for 4 epidemic-prone diseases (i.e., cholera, measles, meningitis, and yellow fever), several general surveillance questions are also included in the tool. The data collected will inform Epi-Detecte strategies to effectively improve disease surveillance in Guinea and serve as a baseline for determining the impact of planned Epi-Detecte interventions on basic surveillance system functions.

Interviewer Guidance:

- The interviewer should contact the DPLM in advance to arrange a site visit with a DPLM official who is knowledgeable about disease surveillance.
- At the beginning of the site visit, the interviewer should explain to the official and to any other persons assisting with data collection (i.e., the interviewee(s)) the purpose of this activity and how the data will be used (see paragraph above). The interviewer should assure the interviewee(s) that they will not be personally identified in the results.
- During the site visit, the interviewer should complete each question in this data collection tool by interviewing the DPLM official and asking for his/her assistance in collecting the requested information and materials. The interviewer should attempt to complete the tool in a single site visit. However, if needed, a follow-up visit could be arranged within 1 week of the initial visit to complete data collection.
- The interviewer should use the "Checklist of materials" at the bottom of this page to ensure that, in addition to completing the questions, all other requested and available materials are obtained.
- Additional interviewer guidance and instruction can be found throughout the document in italics.
- Note that throughout the document, meningococcal meningitis is referred to as meningitis.

Acronyms and Definitions:

DPLM = Division of the Prevention and Control of Disease
DPS = Prefectural Health Directorate (district level)
IDSR = Integrated Disease Surveillance and Response
MOH = Ministry of Health
Priority diseases = African public health priorities, as specified by the IDSR framework and MOHs, as diseases which are largely preventable with well-known and efficacious responses
Epidemic-prone diseases = Subset of priority diseases with a high epidemic potential to cause serious public health impact due to their ability to spread rapidly internationally
Outbreak = Occurrence of cases of disease in excess of what would normally be expected in a defined community, geographic area, or season

Checklist of materials for interviewer to obtain (either copy or take pictures), if available:

☐ Standard case definitions for priority diseases (question 2)
☐ Surveillance feedback from DPLM to DPS (question 9)
☐ Data analyses for priority diseases (question 10)

Please ask the interviewee(s) the following questions and request assistance in collecting the information, as indicated. If needed, use the "Comments" space after each question to describe additional relevant detail and/or challenges in obtaining the information.

Background

1. What is the organizational structure of the DPLM? *(Please describe briefly and/or make copy/take pictures of organizational chart.)*

   ____
   ____
   ____
   ____
   ____
   ____
   ____

General Surveillance and Reporting

2. *Ask to see the standard case definitions for priority diseases (including epidemic-prone diseases) that are used by DPLM staff and, if available, request copies and/or take pictures of these standard case definitions*.

   Are standard case definitions for priority diseases (including epidemic-prone diseases) available at DPLM for staff to reference? *(Check only one)*

   ☐ Yes
   ☐ No
   ☐ Not sure (*explain in comments*)
   Comments: ____

3. *Ask to see the registry where DPLM records the routine weekly reporting of epidemic-prone diseases from each DPS.*

   Are the routine weekly reports of epidemic-prone diseases from each DPS recorded in a registry at DPLM?

   ☐ Yes
   ☐ No
   ☐ Not sure *(explain in comments)*
   Comments: ____

4. *In consultation with the interviewee(s) and using the DLPM registry and/or other available documentation, complete the following table of routine* *<u>weekly reporting</u>* *by month of epidemic-prone diseases from the* ***<u>Boffa DPS</u>*** *for the time period* ***<u>October 1, 2015–March 31, 2016.</u>***
For each week, determine:
1) If the weekly report from the Boffa DPS was received at all ("Received")
If YES, place an "X" in the designated box.
If NO, place a "0" in the designated box.
2) If the weekly report from the Boffa DPS was received on-time ("On-time")
If YES, place an "X" in the designated box.
If NO, place a "0" in the designated box.
**Note that a 5th week is included for each month but may not be needed, depending on the reporting schedule. If not needed, leave the 5th week box(es) blank.*

	**Boffa DPS: Weekly Reporting of Epidemic-prone Diseases**																													
	**October 2015**					**November 2015**					**December 2015**					**January 2016**					**February 2016**					**March 2016**				
**Week of Month**	**1**	**2**	**3**	**4**	**5***	**1**	**2**	**3**	**4**	**5***	**1**	**2**	**3**	**4**	**5***	**1**	**2**	**3**	**4**	**5***	**1**	**2**	**3**	**4**	**5***	**1**	**2**	**3**	**4**	**5***
**Received**																														
**On-time**																														

Comments: ____
____

5. *In consultation with the interviewee(s) and using the DPLM registry and/or other available documentation, determine the method(s) by which the routine* *<u>weekly reports</u>* *were received by DPLM from the* ***<u>Boffa DPS</u>*** *during the time period* ***<u>October 1, 2015–March 31, 2016</u>****. (Check all that apply)*
_____Telephone
_____Fax
_____Text
_____Email
_____Other (*describe*:____)

6. What method of weekly reporting from district DPSs is or would be most helpful to DPLM? *(Please describe)*
____
____
____
____

7. *In consultation with the interviewee(s) and using the DPLM registry and other available documentation, including case investigation forms, complete the following case count table for the* ***Boffa DPS*** *of 4 epidemic-prone diseases for the time period* ***October 1, 2015–March 31, 2016***.

**Boffa DPS**				
**Disease**	**Number of Reported Cases***	**Number of Reported Cases* with Confirmatory Laboratory Testing *Performed***	**Number of Reported Cases with Confirmed Laboratory Test *Results***	**Total Number of Reported Cases* With a Completed Case Investigation Form**
5a1. Cholera				
5b1. Measles				
5c1. Meningitis				
5d1. Yellow fever				

*Total number of cases, including confirmed and suspected cases

Comments: ____
____

Surveillance Feedback

8. Did DPLM provide surveillance feedback (e.g., phone call acknowledging receipt of report, information on data quality, data summary) to any district DPS during **October 1, 2015–March 31, 2016**? *(Check only one)*
   ☐ Yes
   ☐ No
   ☐ Not sure (*explain in comments*)

Comments: ____

9. How did DPLM provide surveillance feedback to <u>any</u> district DPS during **<u>October 1, 2015–March 31, 2016</u>**?
(Check all that apply and <u>request copies and/or take pictures, if available</u>)
_____Not applicable – no surveillance feedback was provided
_____Written report
_____Verbal report
_____Other (*please describe*: ____)

<u>Analysis</u>

10. *Ask to review analyses of DPLM surveillance data for priority diseases* from any district. In consultation with the interviewee(s), complete the following table to characterize the analysis activities at DPLM during* ***<u>October 1, 2015–March 31, 2016</u>****, and <u>request copies and/or take pictures of analyses if available</u>.*

**Activity**	**Answer** (circle one per question)	**Position/Title of the Person Who Completed the Analysis**	**What tools** were used to complete the analysis?**	**Comments**
Plotted the numbers of cases on a graph	Yes No			
Plotted the distribution of cases on a map	Yes No			
Prepared summaries describing cases by characteristics (e.g., age, sex, education level, vaccination status, occupation or place of employment)	Yes No			

*Analyses for any priority diseases, including but not limited to epidemic-prone diseases
**Examples: pen and paper, calculator, computer

Comments: ____
____

11. In what way(s) could Epi-Detecte best help to strengthen the regular review and analysis of weekly-reported surveillance data?

Please provide any other comments below.

End of data collection tool
