# Supplementary material for: Surveillance system assessment in Guinea: Training needed to strengthen data quality and analysis, 2016
Source: PLoS One. 2020 Jun 25;15(6):e0234796. doi: 10.1371/journal.pone.0234796 (PMC7316275; doi:10.1371/journal.pone.0234796)
Supplement: S2 File — (PDF) [file pone.0234796.s002.pdf]

Surveillance des maladies à Boffa, Guinée Outil de collecte de données : Centre de soins de santé

Nom du centre de soins de santé :________________________________

Nom de l'intervieweur : _________________________________
Date :________________________________

Cet outil sera utilisé pour collecter des données de base auprès des centres sanitaires à Boffa, en Guinée portant sur la surveillance de la santé publique, y compris la surveillance basée sur les cas-indicateurs et la surveillance à l'échelle communautaire. Bien que l'accent soit mis sur la surveillance des maladies à tendance épidémique 4 (à savoir, le choléra, la rougeole, la méningite et la fièvre jaune), pendant la période entre le 1er octobre 2015 et le 1er mars 2016, plusieurs questions de surveillance générale sont également incluses dans l'outil. Les données recueillies permettront d'informer les stratégies Epi-Detecte pour améliorer efficacement la surveillance des maladies en Guinée et servir de base pour déterminer l'impact des interventions Epi-Détecte prévues relativement aux fonctions de base du système de surveillance.

Conseils pour l'intervieweur :

- L'intervieweur doit contacter le centre de soins de santé à l'avance pour organiser une visite du site avec la personne désignée en charge de la surveillance au centre de soins de santé (par ex. le responsable de la surveillance des maladies). S'il n'y a pas personne désignée dans ce rôle, le directeur médical du centre de soins de santé peut servir à ce titre.
- Au début de la visite du site, l'intervieweur doit expliquer au responsable de la surveillance des maladies et à toute autre personne aidant à la collecte de données (c.-à-d. la ou les personne(s) interrogée(s)) le but de cette activité et la façon dont les données seront utilisées (voir le paragraphe ci-dessus). L'interviewer doit s'assurer que la ou les personne(s) interrogée(s) ne seront pas personnellement identifiées dans les résultats.
- Au cours de la visite du site, l'intervieweur doit terminer chaque question dans cet outil de collecte de données en interrogeant le responsable de la surveillance des maladies et en demandant son aide pour la collecte des informations et des documents demandés. L'interviewer doit tenter de compléter l'outil avec une seule visite du site. Toutefois, si nécessaire, une visite de suivi pourraient être organisée au cours de la semaine suivant la visite initiale afin de terminer la collecte de données.
- L'intervieweur doit utiliser la « Liste de contrôle des matériaux » au bas de cette page pour veiller à ce que, en plus de répondre aux questions, tous les autres documents demandés et disponibles soient obtenus.
- Vous trouverez de plus amples conseils et directives pour l'interviewer dans le document en italique.
- Veuillez noter que tout le long du document, la méningite à méningocoques est désignée par méningite.

Acronymes et définitions :
DPS = Direction préfectorale sanitaire (niveau de district)
SIMR = Surveillance intégrée de la maladie et riposte
MOH = Ministère de la Santé
Maladies prioritaires = Priorités africaines relatives à la santé publique, telles que spécifiées par le cadre de la SIMR et des MOH ; les maladies qui sont en grande partie évitables avec des ripostes bien connues et efficaces
Maladies épidémiques = Sous-ensemble des maladies prioritaires avec un potentiel épidémique élevé causant des effets graves sur la santé publique en raison de leur capacité à se propager rapidement à l'échelle internationale
Épidémie = Apparition de cas de maladie d'un mombre supérieur à ce à quoi on pourrait s'attendre dans une communauté, une zone géographiques ou

une saison définie.
**Liste de contrôle des matériaux, destinée à l’interviewer (la copier ou la prendre en photo), le cas échéant :**<br>☐ Définition de cas de surveillance communautaire pour les maladies prioritaires (question 3)<br>☐ Définition de cas standard pour les maladies prioritaires (question 4)<br>☐ Commentaires de surveillance de la DPS au centre de soins de santé (question 11)<br>☐ Information de surveillance du centre de soins de santé à la communauté (question 14)<br>☐ Analyse des données pour les maladies prioritaires (question 15)

Veuillez poser les questions suivantes à la ou aux personne(s) interrogée(s) et demandez de l’aide pour la collecte de l’information, comme indiqué. Si nécessaire, utilisez l’espace « Commentaires » après chaque question pour décrire tous les détails pertinents ou les difficultés que vous avez rencontrées pour obtenir les informations.

<u>Contexte</u>

1. Quel est le titre et le rôle de la personne désignée responsable de la surveillance des maladies à l'échelle du centre de soins de santé (par ex. responsable de la surveillance des maladies) ?
   ____
   ____

<u>Surveillance des maladies à l'échelle de la communauté</u>

2. Quel est le titre et le rôle de la personne désignée responsable de la surveillance à l'échelle de la communauté en association avec le centre de soins de santé ?
   ____
   ____

3. *Demandez à voir les définitions de cas de surveillance des maladies prioritaires à l'échelle de la communauté (y compris les maladies à tendance épidémique) et, le cas échéant, <u>demandez des copies ou prenez des photos de ces définitions de cas de surveillance communautaire.</u>*
   Les définitions de surveillance des maladies prioritaires à l'échelle de la communauté (y compris les maladies à tendance épidémique) sont-elles disponibles au centre de soins de santé pour que le personnel puisse les consulter ? *(Cochez une seule réponse)*
   ☐ Oui
   ☐ Non
   ☐ Pas certain (*Veuillez préciser*)
   Commentaires :
   ____

<u>Surveillance des maladies basées au centre de soins de santé</u>

4. *Demandez à voir les définitions de cas standard pour les maladies prioritaires (y compris les maladies à tendance épidémique) qui sont utilisées par le personnel du centre de soins de santé et, le cas échéant, <u>demandez des copies ou prenez des photos de ces définitions de cas standard.</u>*
   Les définitions de cas communautaires pour les maladies prioritaires (y compris les maladies à tendance épidémique) sont-elles disponibles à la DPS pour que le personnel puisse les consulter ? *(Cochez une seule réponse)*
   ☐ Oui
   ☐ Non
   ☐ Pas certain (*Veuillez préciser*)
   Commentaires :
   ____

5. *Demandez à voir le registre des cas du centre de soins de santé dans lesquels les diagnostics de maladies prioritaires (y compris les maladies à tendance épidémique) sont enregistrés.*
   Les diagnostics des maladies prioritaires (y compris les maladies à tendance épidémique) sont-ils enregistrés dans un registre de cas au centre de soins de santé conformément à une définition de cas standard ? *(Cochez une seule réponse)*
   ☐ Oui
   ☐ Non
   ☐ Pas certain (*Veuillez préciser*)
   Commentaires :
   ____

6. *Examinez les entrées dans le registre de cas, le cas échéant.*
   Est-ce que le registre de cas du centre de soins de santé semble être à jour ? *(Cochez une seule réponse)*
   ☐ Non applicable - il n'y a pas de registre de cas
   ☐ Oui
   ☐ Non
   ☐ Pas certain (*Veuillez préciser*)
   Commentaires :
   ____
   ____

7. *En consultation avec la ou les personne(s) interrogée(s) et en utilisant le registre de cas du centre de soins de santé ou toute autre documentation disponible, veuillez remplir le tableau de nombre de cas au centre de soins de santé des 4 maladies à tendance épidémique pour la période du* ***1er octobre 2015 au 31 mars 2016****.*

**Maladie**	**Nombre de Cas**
Choléra	
Rougeole	
Méningite	
Fièvre jaune	

Rapports et commentaires

8. *Demandez si le centre de soins de santé documente le signalement requis immédiat des maladies à tendance épidémique à la DPS, et dans l'affirmative, demandez à voir cette documentation.*
   Est-ce que le centre de soins de santé documente le signalement requis immédiat des maladies à tendance épidémique à la DPLM, entre le **1er octobre 2015 et le 31 mars 2016** ? *(Cochez une seule réponse)*
   ☐ Oui, il y a des documents indiquant que tous les cas de maladies à tendance épidémique ont été immédiatement déclarés à la DPS pendant cette période. (*Décrire la méthode de documentation* : ______________________________)
   ☐ Oui, il y a des documents indiquant que quelques cas de maladies à tendance épidémique ont été immédiatement déclarés à la DPS pendant cette période. (*Décrire la méthode de documentation* : ______________________________)
   ☐ Non, il n'y a aucun document indiquant que les cas de maladies à tendance épidémique ont été immédiatement déclarés à la DPS pendant cette période.
   ☐ Pas certain (*Veuillez préciser*)

Commentaires :

____

9. *Demandez si le centre de soins de santé documente le* <u>*rapport hebdomadaire*</u> *de routine des des maladies à tendance épidémique à la DPS, et dans l'affirmative, demandez à voir cette documentation.*
   Est-ce que le centre de soins de santé documente le rapport <u>hebdomadaire de routine</u> des maladies à tendance épidémique à la DPLM, entre le **<u>1er octobre 2015 et le 31 mars 2016</u>** ? *(Cochez une seule réponse)*
   ☐ Oui, il y a des documents indiquant que <u>tous</u> les rapports hebdomadaires de routine des maladies à tendance épidémique ont été immédiatement déclarés à la DPS pendant cette période. (*Décrire la méthode de documentation* : ____)
   ☐ Oui, il y a des documents indiquant que <u>quelques</u> rapports hebdomadaires de routine des maladies à tendance épidémique ont été immédiatement déclarés à la DPS pendant cette période. (*Décrire la méthode de documentation :* ____)
   ☐ Non, il n'y a aucun document indiquant que les rapports hebdomadaires de routine des maladies à tendance épidémique ont été immédiatement déclarés à la DPS pendant cette période.
   ☐ Pas certain (*Veuillez préciser*)

Commentaires : ____

10. Le centre de soins de santé a-t-il reçu des commentaires de surveillance (par ex., appel téléphonique accusant réception du rapport, d'informations sur la qualité des données, d'un résumé des données) de la DPS entre le **<u>1er octobre 2015 et le 31 mars 2016</u>** ? *(Cochez une seule réponse)*
   ☐ Oui
   ☐ Non
   ☐ Pas certain (*Veuillez préciser*)

Commentaires : ____

11. Comment la DPS a-t-elle fourni des commentaires de surveillance au centre de soins de santé entre le **<u>1er octobre 2015 et le 31 mars 2016</u>** ?
   (Cochez toutes les cases qui s'appliquent et d<u>emandez des copies ou prenez des photos,</u> le cas échéant)
   _____Non applicable –aucun commentaire de surveillance n'a été fourni
   _____Rapport écrit
   _____Rapport oral
   _____Autre (*veuillez décrire* : ____)

12. Quels sont les commentaires de surveillance de la DPS qui sont ou seraient les plus utiles au centre de soins de santé ? *(Veuillez décrire)*

13. Le centre de soins de santé a-t-il fourni des informations de surveillance (par ex., sommaire de données, alertes ou notifications relatives à la santé publique) à la communauté entre le **1er octobre 2015 et le 31 mars 2016** ? *(Cochez une seule réponse)*

☐ Oui
☐ Non
☐ Pas certain (*Veuillez préciser*)

Commentaires :

____

14. Comment le centre de soins de santé a-t-il fourni des commentaires de surveillance à la communauté entre le **1er octobre 2015 et le 31 mars 2016** ?
(Cochez toutes les cases qui s'appliquent et demandez des copies ou prenez des photos, le cas échéant)

_____Non applicable –aucune information de surveillance n'a été fournie
_____Réunion(s) ou forum(s) public(s)
_____Réunion(s) ou forum(s) d'agents de santé communautaire
_____Médias imprimés
_____Radio
_____Autre (*veuillez décrire* : ____)

Analyse :

15. *Demandez à examiner l'analyse des données de surveillance du centre de soins de santé pour les maladies prioritaires*. En consultation avec la ou les personne(s) interrogée(s), remplissez le tableau ci-dessous pour décrire les activités d'analyse au centre de soins de santé entre le* ***1er octobre 2015 et le 31 mars 2016****, et demandez des copies ou prenez ces photos des analyses si elles sont disponibles.*

**Activité**	**Riposte** *(entourez une réponse par question)*	**Poste/titre de la personne qui a effectué l'analyse**	**Quels outils** ont été utilisés pour effectuer l'analyse ?**	**Commentaires**
Reporté les nombres de cas sur un graphique	Oui Non			
Reporté la répartition des cas sur une carte	Oui Non			
Résumés préparés pour décrire des cas par caractéristiques (par ex., âge, sexe, niveau d'éducation, statut d'immunisation, profession ou lieu de travail)	Oui Non			

* Analyses pour toute maladie prioritaire, y compris mais sans s'y limiter, les maladies à tendance épidémique

**Exemples : crayon et papier, calculatrice, ordinateur

Commentaires :

___

___

16. De quelle(s) manière(s) Epi-détecte pourrait permettre de renforcer l'analyse et l'examen courants des données de surveillance hebdomadaires déclarées ?

___

___

___

Épidémies de maladie

17. *Demandez à voir le registre dans lequel les épidémies de maladies suspectées sont enregistrées.*
    Les flambées épidémiques suspectées sont-elles enregistrées dans un registre au centre de soins de santé ? *(Cochez une seule réponse)*
    - ☐ Oui
    - ☐ Non
    - ☐ Pas certain (*Veuillez préciser*)

    Commentaires :
    ____

18. *Examinez les entrées dans le registre des flambées épidémiques.*
    Le registre des flambées épidémiques semble-t-il à jour ? *(Cochez une seule réponse)*
    - ☐ Non applicable - il n’y a pas de registre d’épidémies
    - ☐ Oui
    - ☐ Non
    - ☐ Pas certain (*Veuillez préciser*)

    Commentaires :
    ____

19. *En consultation avec la ou les personne(s) interrogée(s) et en utilisant le registre des flambées épidémiques ou toute autre documentation disponible, veuillez remplir le tableau de nombre de flambées épidémiques au centre de soins de santé suivant des 4 maladies à tendance épidémique pour la période du **1er octobre 2015 au 31 mars 2016**.*

**Maladie**	**Nombre d’épidémies identifiées**	**Nombre de flambées épidémiques faisant l’objet d’une enquête**	**Nombre de flambées épidémiques déclarées à la DPS**	**Dressez la liste des ressources d’intervention, le cas échéant, qui étaient fournies par le MOH pour les enquêtes sur les flambées épidémiques**
Choléra				
Rougeole				
Méningite				

Fièvre jaune				

Commentaires :

Veuillez fournir tout autre commentaire ci-dessous.

Fin de l’outil de collecte de données

Surveillance des maladies à Boffa, Guinée Outil de collecte de données : DPS

Nom de l'intervieweur : ___________________________
Date :____________________________

Cet outil sera utilisé pour collecter des données de base auprès de la Direction préfectorale sanitaire (DPS) à Boffa, en Guinée portant sur la surveillance de la santé publique, y compris la surveillance basée sur les cas-indicateurs et la surveillance à l'échelle communautaire. Bien que l'accent soit mis sur la surveillance des maladies à tendance épidémique 4 (à savoir, le choléra, la rougeole, la méningite et la fièvre jaune), pendant la période entre le 1er octobre 2015 et le 1er mars 2016, plusieurs questions de surveillance générale sont également incluses dans l'outil. Les données recueillies permettront d'informer les stratégies Epi-Detecte pour améliorer efficacement la surveillance des maladies en Guinée et servir de base pour déterminer l'impact des interventions Epi-Détecte prévues relativement aux fonctions de base du système de surveillance.

<u>Conseils pour l'intervieweur</u>

- L'intervieweur doit contacter la DPS à l'avance pour organiser une visite du site avec un agent de la MCM (Medicine Charge de Maladies). Si cela s'avère impossible, l'intervieweur doit organiser une visite du site avec un agent de la DPS qui connaît bien la surveillance des maladies.
- Au début de la visite du site, l'intervieweur doit expliquer à l'agent de la MCM ou à toute autre personne aidant à la collecte de données (c.-à-d. la ou les personne(s) interrogée(s) le but de cette activité et la façon dont les données seront utilisées (voir le paragraphe ci-dessus). L'intervieweur doit s'assurer que la ou les personne(s) interrogée(s) ne seront pas personnellement identifiées dans les résultats.
- Au cours de la visite du site, l'intervieweur doit terminer chaque question dans cet outil de collecte de données en interrogeant l'agent de la MCM et en demandant son aide pour la collecte des informations et des documents demandés. L'intervieweur doit tenter de compléter l'outil avec une seule visite du site. Toutefois, si nécessaire, une visite de suivi pourraient être organisée au cours de la semaine suivant la visite initiale afin de terminer la collecte de données.
- L'intervieweur doit utiliser la « Liste de contrôle des matériaux » au bas de cette page pour veiller à ce que, en plus de répondre aux questions, tous les autres documents demandés et disponibles soient obtenus.
- Vous trouverez de plus amples conseils et directives pour l'intervieweur dans le document en italique.
- Veuillez noter que tout le long du document, la méningite à méningocoques est désignée par méningite.

Acronymes et définitions :
DPLM = Division de la prévention et de contrôle des maladies
DPS = Direction préfectorale sanitaire (niveau de district)
SIMR = Surveillance intégrée de la maladie et riposte
MCM = Médecine chargée des maladies
MOH = Ministère de la Santé
Maladies prioritaires = Priorités africaines relatives à la santé publique, telles que spécifiées par le cadre de la SIMR et des MOH ; les maladies qui sont en grande partie évitables avec des ripostes bien connues et efficaces
Maladies épidémiques = Sous-ensemble des maladies prioritaires avec un potentiel épidémique élevé causant des effets graves sur la santé publique en raison de leur capacité à se propager rapidement à l'échelle internationale
Épidémie = Apparition de cas de maladie d'un mombre supérieur à ce à quoi on pourrait s'attendre dans une communauté, une zone géographiques ou une saison définie.

Liste de contrôle des matériaux destinée à l'intervieweur (la copier ou la prendre en photo), le cas échéant :
☐ Définition de cas de surveillance communautaire pour les maladies prioritaires (question 2)
☐ Définition de cas standard pour les maladies prioritaires (question 3)
☐ Commentaires de surveillance de la DPLM à la DPS (question 12)
☐ Commentaires de surveillance de la DPS aux centres sanitaires de Boffa ou à l'hôpital départemental (question 15)
☐ Analyse des données pour les maladies prioritaires (question 16)

Veuillez poser les questions suivantes à la ou aux personne(s) interrogée(s) et demandez de l'aide pour la collecte de l'information, comme indiqué. Si nécessaire, utilisez l'espace « Commentaires » après chaque question pour décrire tous les détails pertinents ou les difficultés que vous avez rencontrées pour obtenir les informations.

<u>Surveillance des maladies à l'échelle de la communauté</u>

1. Quel est le titre et le rôle de la personne désignée responsable de la surveillance à l'échelle de la communauté en association avec la DPS ?
   (S'il n'y a personne à la DPS qui occupe cette fonction, sveuillez indiquer « non applicable » dans l'espace ci-dessous.)
   ____
   ____

2. *Demandez à voir les définitions de cas de surveillance des maladies prioritaires à l'échelle de la communauté (y compris les maladies à tendance épidémique) et, le cas échéant, <u>demandez des copies ou prenez des photos de ces définitions de cas de surveillance communautaire.</u>*
   Les définitions de surveillance des maladies prioritaires à l'échelle de la communauté (y compris les maladies à tendance épidémique) sont-elles disponibles à la DPS pour que le personnel puisse les consulter ? *(Cochez une seule réponse)*
   ☐ Oui
   ☐ Non
   ☐ Pas certain (*Veuillez préciser*)
   Commentaires :
   ____

<u>Surveillance générale et rapports</u>

3. *Demandez à voir les définitions de cas standard pour les maladies prioritaires (y compris les maladies à tendance épidémique) qui sont utilisées par le personnel de la DPS et, le cas échéant, <u>demandez des copies ou prenez des photos de ces définitions de cas standard.</u>*
   Les définitions de cas standard pour les maladies prioritaires (y compris les maladies à tendance épidémique) sont-elles disponibles à la DPS pour que le personnel puisse les consulter ?
   (Cochez une seule réponse)

☐ Oui
☐ Non
☐ Pas certain (*Veuillez préciser*)
Commentaires :

---

4. *Demandez à voir le registre où la DPS enregistre le rapport <u>hebdomadaire de routine</u> des maladies à tendance épidémique des centres sanitaires et de l'hôpital départemental de Boffa.*
   Les rapports de routine hebdomadaire des maladies à tendance épidémique de chaque centre de soins de santé et de l'hôpital départemental de Boffa sont-ils enregistrés dans un registre à la DPS ?
   (Cochez une seule réponse)
   ☐ Oui
   ☐ Non
   ☐ Pas certain (*Veuillez préciser*)
   Commentaires :
   ____

5. *En consultation avec la ou les personne(s) interrogée(s) et en utilisant le registre ou toute autre documentation disponible, remplissez les tableaux suivants des rapports de routine hebdomadaire par mois de maladies à tendance épidémique de la DPS, provenant des centres sanitaires et de l'hôpital départemental de Boffa pour la période du **1er octobre 2015 au 31 mars 2016**. (Voir les tableaux sur les 2 pages suivantes)*
   Pour chaque semaine, veuillez déterminer :
   1) *Si le rapport hebdomadaire de l'installation a bien été reçu (« Reçu »)*
      Si OUI, placez un « X » dans la zone désignée.
      Si NON, placez un « 0 » dans la zone désignée.
   2) *Si le rapport hebdomadaire de l'installation a été reçu dans les temps (« À temps »)*
      Si OUI, placez un « X » dans la zone désignée.
      Si NON, placez un « 0 » dans la zone désignée.

   **Noter qu'une 5e semaine est incluse pour chaque mois, mais il est possible que ce ne soit pas nécessaire, selon le calendrier des rapports. Si ce n'est pas nécessaire, laissez la ou les case(s) de la 5e semaine vierge(s).*

Mois	Semaine du mois	Rapports de situation	Installation établissant les rapports								
			Colia	Mankountan	Koba	Lisso	Tamita	Douprou	Tougnify	Boffe Centre	Hôpital départemental
Octobre 2015	1	Reçu									
		Dans les délais									
	2	Reçu									
		Dans les délais									
	3	Reçu									
		Dans les délais									
	4	Reçu									
		Dans les délais									
	5*	Reçu									
		Dans les délais									
Novembre 2015	1	Reçu									
		Dans les délais									
	2	Reçu									
		Dans les délais									
	3	Reçu									
		Dans les délais									
	4	Reçu									
		Dans les délais									
	5*	Reçu									
		Dans les délais									
Décembre 2015	1	Reçu									
		Dans les									

		délais									
	2	Reçu									
		Dans les délais									
	3	Reçu									
		Dans les délais									
	4	Reçu									
		Dans les délais									
	5*	Reçu									
		Dans les délais									
**Mois**	**Semaine du mois**	**Rapports de situation**	**Installation établissant les rapports**								
			**Colia**	**Mankountan**	**Koba**	**Lisso**	**Tamita**	**Douprou**	**Tougnify**	**Boffe Centre**	**Hôpital départemental**
**Janvier 2016**	1	Reçu									
		Dans les délais									
	2	Reçu									
		Dans les délais									
	3	Reçu									
		Dans les délais									
	4	Reçu									
		Dans les délais									
	5*	Reçu									
		Dans les délais									
**Février 2016**	1	Reçu									
		Dans les délais									
	2	Reçu									
		Dans les									

		**délais**									
	**3**	**Reçu**									
		**Dans les délais**									
	**4**	**Reçu**									
		**Dans les délais**									
	**5***	**Reçu**									
		**Dans les délais**									
**Mars 2016**	**1**	**Reçu**									
		**Dans les délais**									
	**2**	**Reçu**									
		**Dans les délais**									
	**3**	**Reçu**									
		**Dans les délais**									
	**4**	**Reçu**									
		**Dans les délais**									
	**5***	**Reçu**									
		**Dans les délais**									

6. *En consultation avec la ou les personne(s) interrogée(s) et en utilisant le registre ou d'autres documents disponibles, veuillez déterminer la ou les méthode(s) par lesquelles les rapports hebdomadaires de routine ont été reçus par la DPS de chaque installation établissant les rapports pendant la période du **1er octobre 2015 au 31 mars 2016**.*
Pour chaque installation établissant les rapports, placez un « X » dans le(s) case(s) appropriée(s), en indiquant toutes les méthodes qui s'appliquent.

**Méthode d'établissement de rapports à la DPS**	**Installation établissant les rapports**								
	**Colia**	**Mankountan**	**Koba**	**Lisso**	**Tamita**	**Douprou**	**Tougnify**	**Boffe Centre**	**Hôpital départemental**
Téléphone									
Fax									
Texte									
Email									
Autre (décrire) :									

7. Quelle est la méthode de rapport hebdomadaire des installations qui est ou serait la plus utile à la DPS ? *(Veuillez décrire)*
___
___
___
___

8. *En consultation avec la ou les personne(s) interrogée(s) et en utilisant le registre ou toute autre documentation disponible, veuillez remplir les tableaux de nombre de cas suivants par le centre de soins de santé et l'hôpital départemental de Boffa, des 4 maladies à tendance épidémique pour la période du **1er octobre 2015 au 31 mars 2016**.*

**Installation établissant les rapports et maladie**	**Nombre de cas déclarés***	**Nombre de cas déclarés* avec des tests de confirmation de diagnostic *effectués***	**Nombre de cas déclarés avec les *résultats* des tests de confirmation de diagnostic**	**Nombre total de cas déclarés* Analysé par la DPS**	**Nombre total de cas déclarés* Avec un formulaire d'enquête de cas rempli**
**Colia**					
Choléra					
Rougeole					
Méningite					
Fièvre jaune					
**Mankountan**					
Choléra					
Rougeole					
Méningite					
Fièvre jaune					
**Koba**					
Choléra					
Rougeole					
Méningite					
Fièvre jaune					

*Nombre total de cas, y compris les cas confirmés et suspectés

Commentaires :

___

**Installation établissant les rapports et maladie**	**Nombre de cas déclarés***	**Nombre de cas déclarés* avec des tests de confirmation de diagnostic *effectués***	**Nombre de cas déclarés avec les *résultats* des tests de confirmation de diagnostic**	**Nombre total de cas déclarés* Analysé par la DPS**	**Nombre total de cas déclarés* Avec un formulaire d'enquête de cas rempli**
**Lisso**					
Choléra					
Rougeole					
Méningite					
Fièvre jaune					
**Tamita**					
Choléra					
Rougeole					
Méningite					
Fièvre jaune					
**Douprou**					
Choléra					
Rougeole					
Méningite					
Fièvre jaune					

*Nombre total de cas, y compris les cas confirmés et suspectés
Commentaires :

**Installation établissant les rapports et maladie**	**Nombre de cas déclarés***	**Nombre de cas déclarés* avec des tests de confirmation de diagnostic *effectués***	**Nombre de cas déclarés avec les *résultats* des tests de confirmation de diagnostic**	**Nombre total de cas déclarés* Analysé par la DPS**	**Nombre total de cas déclarés* Avec un formulaire d'enquête de cas rempli**
**Tougnify**					
Choléra					
Rougeole					
Méningite					
Fièvre jaune					
**Boffe Centre**					
Choléra					
Rougeole					
Méningite					
Fièvre jaune					
**Hôpital départemental**					
Choléra					
Rougeole					
Méningite					
Fièvre jaune					

*Nombre total de cas, y compris les cas confirmés et suspectés

Commentaires :

9. *Demandez si la DPS documente le signalement requis* *__immédiat__* *des maladies à tendance épidémique à la DPLM, et dans l'affirmative, demandez à voir cette documentation.*
Est-ce que la DPS documente le signalement requis <u>immédiat</u> des maladies à tendance épidémique à la DPLM, entre le **<u>1er octobre 2015 et le 31 mars 2016</u>** ? *(Cochez une seule réponse)*
☐ Oui, il y a des documents indiquant que <u>tous</u> les cas de maladies à tendance épidémique ont été immédiatement déclarés à la DPLM pendant cette période. (*Décrire la méthode de documentation* : ____)
☐ Oui, il y a des documents indiquant que <u>quelques</u> cas de maladies à tendance épidémique ont été immédiatement déclarés à la DPLM pendant cette période. (*Décrire la méthode de documentation* : ____)
☐ Non, il n'y a aucun document indiquant que les cas de maladies à tendance épidémique ont été immédiatement déclarés à la DPLM pendant cette période.
☐ Pas certain (*Veuillez préciser*)

Commentaires :

____

10. *Demandez si la DPS documente le rapport* *__hebdomadaire de routine__* *des maladies à tendance épidémique à la DPLM, et dans l'affirmative, demandez à voir cette documentation.*
Est-ce que la DPS documente l<u>e rapport hebdomadaire</u> de routine des maladies à tendance épidémique à la DPLM, entre le **<u>1er octobre 2015 et le 31 mars 2016</u>** ? *(Cochez une seule réponse)*
☐ Oui, il y a des documents indiquant que <u>tous</u> les rapports hebdomadaires de routine des maladies à tendance épidémique ont été immédiatement déclarés à la DPLM pendant cette période. (*Décrire la méthode de documentation* : ____)
☐ Oui, il y a des documents indiquant que <u>quelques</u> rapports hebdomadaires de routine des maladies à tendance épidémique ont été immédiatement déclarés à la DPLM pendant cette période. (*Décrire la méthode de documentation :* ____)
☐ Non, il n'y a aucun document indiquant que les rapports hebdomadaires de routine des maladies à tendance épidémique ont été immédiatement déclarés à la DPLM pendant cette période.
☐ Pas certain (*Veuillez préciser*)

Commentaires :

____

<u>Commentaire de surveillance</u>

11. La DPS a-t-elle reçu des commentaires de surveillance (par ex., appel téléphonique accusant réception du rapport, d'informations sur la qualité des données, d'un résumé des données) de la DPLM entre le **1er octobre 2015 et le 31 mars 2016** ?
    (Cochez une seule réponse)
    - ☐ Oui
    - ☐ Non
    - ☐ Pas certain (*Veuillez préciser*)

Commentaires :

12. Comment la DPLM a-t-elle fourni des commentaires de surveillance à la DPS entre le **1er octobre 2015 et le 31 mars 2016** ?
(Cochez toutes les cases qui s'appliquent et demandez des copies ou prenez des photos, le cas échéant)
_____Non applicable –aucun commentaire de surveillance n'a été fourni
_____Rapport écrit
_____Rapport oral
_____Autre (*veuillez décrire* : ____)

13. Quels sont les commentaires de surveillance de la DPLM qui sont ou seraient les plus utiles à la DPS ? *(Veuillez décrire)*
____
____
____
____

14. La DPS a-t-elle fourni des commentaires de surveillance (par ex., appel téléphonique accusant réception du rapport, d'informations sur la qualité des données, d'un résumé des données) aux centres sanitaires ou à l'hôpital départemental de Boffa entre le **1er octobre 2015 et le 31 mars 2016** ?
(Cochez une seule réponse)
☐ Oui
☐ Non
☐ Pas certain (*Veuillez préciser*)

Commentaires :
____

15. Comment la DPS a-t-elle fourni des commentaires de surveillance aux centres sanitaires ou à l'hôpital départemental de Boffa entre le **1er octobre 2015 et le 31 mars 2016** ?
(Cochez toutes les cases qui s'appliquent et demandez des copies ou prenez des photos, le cas échéant)
_____Non applicable –aucun commentaire de surveillance n'a été fourni
_____Rapport écrit
_____Rapport oral
_____Autre (*veuillez décrire* : ____)

<u>Analyse :</u>

16. *Demandez à examiner l'analyse des données de surveillance de la DPS pour les maladies prioritaires*. En consultation avec la ou les personne(s) interrogée(s), remplissez le tableau ci-dessous pour décrire les activités d'analyse à la DPS entre le **<u>1er octobre 2015 et le 31 mars 2016</u>**, et <u>demandez des copies ou prenez ces photos des analyses si elles sont disponibles.</u>*

**Activité**	**Riposte** *(entourez une réponse par question)*	**Poste/titre de la personne qui a effectué l'analyse**	**Quels outils** ont été utilisés pour effectuer l'analyse ?**	**Commentaires**
Reporté les nombres de cas sur un graphique	Oui Non			
Reporté la répartition des cas sur une carte	Oui Non			
Résumés préparés pour décrire des cas par caractéristiques (par ex., âge, sexe, niveau d'éducation, statut d'immunisation, profession ou lieu de travail)	Oui Non			

* Analyses pour toute maladie prioritaire, y compris mais sans s'y limiter, les maladies à tendance épidémique

**Exemples : crayon et papier, calculatrice, ordinateur

Commentaires :

____

17. De quelle(s) manière(s) Epi-détecte pourrait permettre de renforcer l'analyse et l'examen courants des données de surveillance hebdomadaires déclarées ?

____
____
____
____
____
____

Veuillez fournir tout autre commentaire ci-dessous.

Fin de l'outil de collecte de données

Surveillance des maladies à Boffa, Guinée **Outil de collecte de données DPLM**

Nom de l'interviewer : ____________________________
Date :_____________________________

Cet outil sera utilisé pour collecter des données de base auprès de la Division de la prévention et de contrôle des maladies (DPLM) en Guinée portant sur la surveillance de la santé publique, y compris la surveillance basée sur les cas- indicateurs et la surveillance à l'échelle communautaire entre le 1er octobre 2015 et le 1er mars 2016 à Boffa, en Guinée. Bien que l'accent soit mis sur la surveillance des maladies à tendance épidémique 4 (à savoir, le choléra, la rougeole, la méningite et la fièvre jaune), plusieurs questions de surveillance générale sont également incluses dans l'outil. Les données recueillies permettront d'informer les stratégies Epi-Detecte pour améliorer efficacement la surveillance des maladies en Guinée et servir de base pour déterminer l'impact des interventions Epi-Détecte prévues relativement aux fonctions de base du système de surveillance.

<u>Conseils pour l'intervieweur</u>

- L'intervieweur doit contacter la DPLM à l'avance pour organiser une visite du site avec un agent de la DPLM qui connaît bien la surveillance des maladies.
- Au début de la visite du site, l'intervieweur doit expliquer à l'agent et à toute autre personne aidant à la collecte de données (c.-à-d. la ou les personne(s) interrogée(s)) le but de cette activité et la façon dont les données seront utilisées (voir le paragraphe ci-dessus). L'interviewer doit s'assurer que la ou les personne(s) interrogée(s) ne seront pas personnellement identifiées dans les résultats.
- Au cours de la visite du site, l'intervieweur doit terminer chaque question dans cet outil de collecte de données en interrogeant l'agent DPLM et en demandant son aide pour la collecte des informations et des documents demandés. L'interviewer doit tenter de compléter l'outil avec une seule visite du site. Toutefois, si nécessaire, une visite de suivi pourraient être organisée au cours de la semaine suivant la visite initiale afin de terminer la collecte de données.
- L'intervieweur doit utiliser la « Liste de contrôle des matériaux » au bas de cette page pour veiller à ce que, en plus de répondre aux questions, tous les autres documents demandés et disponibles soient obtenus.
- Vous trouverez de plus amples conseils et directives pour l'interviewer dans le document en italique.
- Veuillez noter que tout le long du document, la méningite à méningocoques est désignée par méningite.

Acronymes et définitions :
DPLM = Division de la prévention et de contrôle des maladies
DPS = Direction préfectorale sanitaire (niveau de district)
SIMR = Surveillance intégrée de la maladie et riposte
MOH = Ministère de la Santé
Maladies prioritaires = Priorités africaines relatives à la santé publique, telles que spécifiées par le cadre de la SIMR et des MOH, comme étant des maladies qui sont en grande partie évitables avec des ripostes bien connues et efficaces
Maladies épidémiques = Sous-ensemble des maladies prioritaires avec un potentiel épidémique élevé causant des effets graves sur la santé publique en raison de leur capacité à se propager rapidement à l'échelle internationale
Épidémie = Apparition de cas de maladie d'un nombre supérieur à ce à quoi on pourrait s'attendre dans une communauté, une zone géographiques ou une saison définie.

Liste de contrôle des matériaux, destinée à l'intervieweur (la copier ou la prendre en photo), le cas échéant :
☐ Définition de cas standard pour les maladies prioritaires (question 2)
☐ Commentaires de surveillance de la DPLM à la DPS (question 9)
☐ Analyse des données pour les maladies prioritaires (question 10)

Veuillez poser les questions suivantes à la ou aux personne(s) interrogée(s) et demandez de l'aide pour la collecte de l'information, comme indiqué. Si nécessaire, utilisez l'espace « Commentaires » après chaque question pour décrire tous les détails pertinents ou les difficultés que vous avez rencontrées pour obtenir les informations.

Contexte

1. Quelle est la structure organisationnelle de la DPLM ? *(Veuillez décrire brièvement et/ou faire une copie/prendre des photos de l'organigramme.)*
   ____
   ____
   ____
   ____
   ____
   ____
   ____

Surveillance générale et rapports

2. *Demandez à voir les définitions de cas standard pour les maladies prioritaires (y compris les maladies à tendance épidémique) qui sont utilisées par le personnel DPLM et, le cas échéant, demandez des copies ou prenez des photos de ces définitions de cas standard.*
   Les définitions de cas standard pour les maladies prioritaires (y compris les maladies à tendance épidémique) sont-elles disponibles à la DPLM pour que le personnel puisse les consulter ?
   (Cochez une seule réponse)
   ☐ Oui
   ☐ Non
   ☐ Pas certain (*Veuillez préciser*)
   Commentaires :
   ____

3. *Demandez à voir le registre où la DPLM enregistre le rapport <u>hebdomadaire de routine</u> des maladies à tendance épidémique de chaque DPS.*

   Les <u>rapports de routine hebdomadaire</u> des maladies à tendance épidémique de chaque DPS sont-ils enregistrés dans un registre à la DPLM ?

   ☐ Oui
   ☐ Non
   ☐ Pas certain (*Veuillez préciser*)

   Commentaires :

   ____

4. *En consultation avec la ou les personne(s) interrogée(s) et en utilisant le registre de la DLPM ou toute autre documentation disponible, remplissez le tableau suivant des rapports de routine hebdomadaire par mois de maladies à tendance épidémique de la* ***DPS à Boffa*** *pour la période du* ***1er octobre 2015 au 31 mars 2016****.*
Pour chaque semaine, veuillez déterminer :
1) Si le rapport hebdomadaire de la DPS de Boffa a bien été reçu (« Reçu »)
Si OUI, placez un « X » dans la zone désignée.
Si NON, placez un « 0 » dans la zone désignée.
2) Si le rapport hebdomadaire de la DPS de Boffa a été reçu dans les temps (« À temps »)
Si OUI, placez un « X » dans la zone désignée.
Si NON, placez un « 0 » dans la zone désignée.
**Noter qu'une 5e semaine est incluse pour chaque mois, mais il est possible que ce ne soit pas nécessaire, selon le calendrier des rapports. Si ce n'est pas nécessaire, laissez la ou les case(s) de la 5e semaine vierge(s).*

	**DPS à Boffa : Rapport hebdomadaire des maladies à tendance épidémique**																													
	**Octobre 2015**					**Novembre 2015**					**Décembre 2015**					**Janvier 2016**					**Février 2016**					**Mars 2016**				
**Semaine du mois**	**1**	**2**	**3**	**4**	**5***	**1**	**2**	**3**	**4**	**5***	**1**	**2**	**3**	**4**	**5***	**1**	**2**	**3**	**4**	**5***	**1**	**2**	**3**	**4**	**5***	**1**	**2**	**3**	**4**	**5***
**Reçu**																														
**Dans les délais**																														

Commentaires : ____
____

5. *En consultation avec la ou les personne(s) interrogée(s) et en utilisant le registre de la DPLM ou d'autres documents disponibles, veuillez déterminer la ou les méthode(s) par lesquelles les rapports hebdomadaires de routine ont été reçus par la DPLM de la* ***DPS à Boffa*** *pendant la période du* ***1er octobre 2015 au 31 mars 2016****.*
(Cochez toutes les cases qui s'appliquent)
_____Téléphone
_____Fax
_____Texte
_____Email
_____Autre (*Veuillez décrire*:____)

6. Quelle est la méthode de rapport hebdomadaire des DPS de district qui est ou serait la plus utile à la DPLM ? *(Veuillez décrire)*
____
____
____
____

7. *En consultation avec la ou les personne(s) interrogée(s) et en utilisant le registre de la DLPM ou toute autre documentation disponible, y compris les formulaires d'enquête de cas, remplissez le tableau de nombre de cas pour la* ***DPS à Boffa*** *de 4 maladies à tendance épidémique pour la période du* ***1er octobre 2015 au 31 mars 2016****.*

DPS à Boffa				
**Maladie**	**Nombre de cas déclarés***	**Nombre de cas déclarés* avec des tests de confirmation de diagnostic *effectués***	**Nombre de cas déclarés avec les *résultats* des tests de confirmation de diagnostic**	**Nombre total de cas déclarés* Avec un formulaire d'enquête de cas rempli**
5a1. Choléra				
5b1. Rougeole				
5c1. Méningite				
5d1. Fièvre jaune				

*Nombre total de cas, y compris les cas confirmés et suspectés

Commentaires :
____
____

Commentaire de surveillance

8. La DPLM a-t-elle fourni des commentaires de surveillance (par ex., appel téléphonique accusant réception du rapport, d'informations sur la qualité des données, d'un résumé des données) à toute DPS de district entre le **1er octobre 2015 et le 31 mars 2016** ? *(Cochez une seule réponse)*
   - ☐ Oui
   - ☐ Non
   - ☐ Pas certain (*Veuillez préciser*)

Commentaires :

9. Comment la DPLM a-t-elle fourni des commentaires de surveillance à toute DPS de district entre le **1er octobre 2015 et le 31 mars 2016** ?
(Cochez toutes les cases qui s'appliquent et demandez des copies ou prenez des photos, le cas échéant)
_____Non applicable –aucun commentaire de surveillance n'a été fourni
_____Rapport écrit
_____Rapport oral
_____Autre (*veuillez décrire* : ____)

Analyse :

10. *Demandez à examiner l'analyse des données de surveillance de la DPLM pour les maladies prioritaires* de tout district. En consultation avec la ou les personne(s) interrogée(s), remplissez le tableau ci-dessous pour décrire les activités d'analyse à la DPLM entre le* ***1er octobre 2015 et le 31 mars 2016****, et demandez des copies ou prenez ces photos des analyses si elles sont disponibles.*

**Activité**	**Riposte** (entourez une réponse par question)	**Poste/titre de la personne qui a effectué l'analyse**	**Quels outils** ont été utilisés pour effectuer l'analyse ?**	**Commentaires**
Reporté les nombres de cas sur un graphique	Oui Non			
Reporté la répartition des cas sur une carte	Oui Non			
Résumés préparés pour décrire des cas par caractéristiques (par ex., âge, sexe, niveau d'éducation, statut d'immunisation, profession ou lieu de travail)	Oui Non			

* Analyses pour toutes les maladies prioritaires, y compris mais sans s'y limiter, les maladies à tendance épidémique
**Exemples : crayon et papier, calculatrice, ordinateur

Commentaires :
____
____

11. De quelle(s) manière(s) Epi-détecte pourrait permettre de renforcer l'analyse et l'examen courants des données de surveillance hebdomadaires déclarées ?

Veuillez fournir tout autre commentaire ci-dessous.

Fin de l'outil de collecte de données
